# The GCKR-P446L gene variant predisposes to raised blood cholesterol and lower blood glucose in the P446L mouse-a model for GCKR rs1260326

**DOI:** 10.1016/j.molmet.2023.101722

**Published:** 2023-04-07

**Authors:** Brian E. Ford, Shruti S. Chachra, Katrina Rodgers, Tabassum Moonira, Ziad H. Al-Oanzi, Quentin M. Anstee, Helen L. Reeves, Jörn M. Schattenberg, Rebecca J. Fairclough, David M. Smith, Dina Tiniakos, Loranne Agius

**Affiliations:** 1 Biosciences Institute, Newcastle University, Newcastle upon Tyne, NE2 4HH, UK; 2 Jouf University, Clinical Laboratory Science, Sakaka, Saudi Arabia; 3 Translational and Clinical Research Institute, Newcastle University, Newcastle upon Tyne, UK; 4 Newcastle NIHR Biomedical Research Center, Newcastle upon Tyne Hospitals NHS Trust, Newcastle upon Tyne, UK; 5 Metabolic Liver Research Programm, Department of Medicine, University Hospital Mainz, Mainz, Germany; 6 Emerging Innovations Unit, Discovery Sciences, BioPharmaceuticals R&D, AstraZeneca, Cambridge, UK; 7 Dept of Pathology, Aretaieion Hospital, Medical School, National and Kapodistrian University of Athens, Athens, Greece

**Keywords:** Liver, Glucose metabolism, Glucokinase, Type 2 diabetes, Fatty liver, Blood cholesterol

## Abstract

**Objectives:**

The Glucokinase Regulatory Protein GKRP, encoded by *GCKR*, enables acute regulation of liver glucokinase to support metabolic demand. The common human *GCKR* rs1260326:Pro446 > Leu variant within a large linkage disequilibrium region associates with pleiotropic traits including lower Type 2 diabetes risk and raised blood triglycerides and cholesterol. Whether the *GCKR-*P446 > L substitution is causal to the raised lipids is unknown. We determined whether mouse GKRP phenocopies the human GKRP:P446 > L substitution and studied a GKRP:P446L knockin mouse to identify physiological consequences to P446 > L.

**Methods:**

GKRP-deficient hepatocytes were transfected with adenoviral vectors for human or mouse GKRP:446 P or 446 L for cellular comprehensive analysis including transcriptomics consequent to P446 > L. Physiological traits in the diet-challenged P446L mouse were compared with pleiotropic associations at the human rs1260326 locus. Transcriptomics was compared in P446L mouse liver with hepatocytes overexpressing glucokinase or GKRP:446 P/L.

**Results:**

1. P446 > L substitution in mouse or human GKRP similarly compromises protein expressivity of GKRP:446 L, nuclear sequestration of glucokinase and counter-regulation of gene expression. 2. The P446L knockin mouse has lower liver glucokinase and GKRP protein similar to human liver homozygous for rs1260326-446 L. 3. The diet-challenged P446L mouse has lower blood glucose, raised blood cholesterol and altered hepatic cholesterol homeostasis consistent with relative glucokinase-to-GKRP excess, but not raised blood triglycerides.

**Conclusions:**

Mouse GKRP phenocopies the human GKRP:P446 > L substitution despite the higher affinity for glucokinase of human GKRP. The diet-challenged P446L mouse replicates several traits found in association with the rs1260326 locus on chromosome 2 including raised blood cholesterol, lower blood glucose and lower liver glucokinase and GKRP protein but not raised blood triglycerides.

## Highlights

• P446 > L substitution in mouse or human GKRP compromises GKRP stability and GK binding.
• The GKRP:P446L mouse has low liver GKRP and glucokinase similar to rs1260326-TT liver.
• The GKRP:P446L mouse manifests low blood glucose and insulin similar to rs160326-TT.
• The diet-challenged GKRP:P446L mouse has high blood cholesterol but not triglyceride.
• The GKRP:P446L mouse replicates the raised blood cholesterol linked to rs1260326(C > T).

## Graphical abstract

**Figure:**
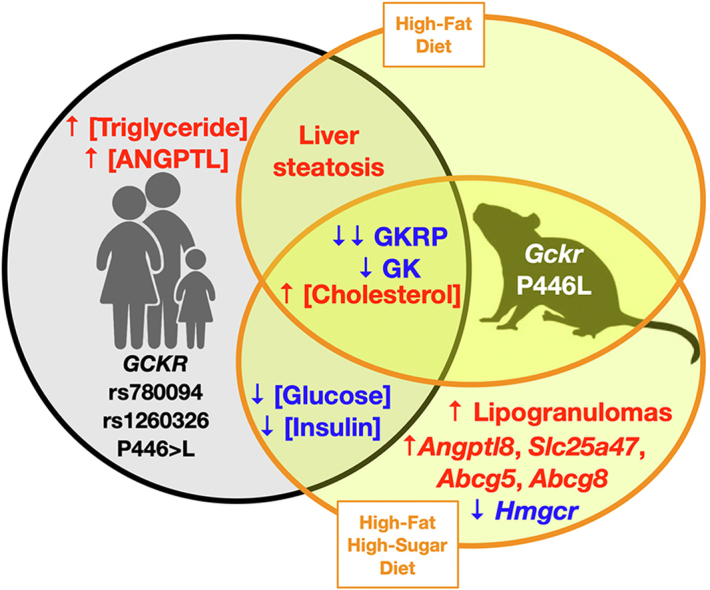

## 1 Introduction

Genome wide studies identified hundreds of gene loci associated with Type 2 diabetes or blood lipids [1,2], each harbouring multiple variants which are a potential resource for exploring disease mechanisms. Several intronic and an exonic (rs1260326^P446>L^) variant in the *GCKR* gene, within a large region of linkage disequilibrium on chromosome-2, associate with raised blood triglycerides and cholesterol and with increased risk for fatty liver disease but with decreased risk for Type 2 diabetes [[3], [4], [5], [6], [7], [8], [9]]. The *GCKR* gene encodes the glucokinase regulatory protein GKRP, which is expressed predominantly in the liver, and localized to the hepatocyte nucleus [10,11]. Converse risks for Type 2 diabetes and liver disease through the same *GCKR* variant has implications for therapies targeting GKRP or glucokinase for type 2 diabetes [12].

The only established function of GKRP is as a negative regulator for glucokinase (GK) which catalyses the first reaction in hepatic glucose metabolism. GKRP belongs to the sugar isomerase family and has a high-affinity binding site for fructose 6-P and fructose 1-P which enhance and attenuate, respectively, its affinity for GK [13,14]. It is a competitive inhibitor of GK with glucose and sequesters GK in the nucleus at basal blood glucose releasing GK to the cytoplasm in response to raised blood glucose after a meal. This allows a reserve GK pool in the nucleus, which can be rapidly mobilised to meet metabolic demand [10,11]. Cytoplasmic GK activity is determined by both the affinity of GKRP for GK and by the molar ratio of GK-to-GKRP, which is dependent on nutritional state [[15], [16], [17]].

The assumed hypothesis for the raised blood and liver lipids and lower blood glucose associated with the *GCKR* locus is that the missense GKRP:446 L variant impairs GK binding to GKRP [[18], [19], [20]] and thereby favours hepatic conversion of glucose to triglyceride through the uninhibited GK [21]. Other hypotheses are that the raised triglycerides may be due to GKRP functions independent of its interaction with GK [14], that the intronic rs780094 variant affects gene transcription [22], or other neighbouring gene variants at the locus may have synergistic effects [23]. Although the first hypothesis is assumed, there has been no supportive evidence from animal models that the *GCKR* P446 > L substitution lowers blood glucose or raises blood triglycerides. Whether the rodent is a valid model for the human GKRP:P446 > L substitution is contentious [19], because of apparent species differences in the P446 > L substitution between human and rat GKRP [18,19,24].

Here we assessed from cellular studies the validity of mouse GKRP to model the human *GCKR*:P446 > L substitution by expressing human or mouse GKRP:446 P/L at physiological GKRP-to-GK ratios in GKRP-deficient mouse hepatocytes and show that mouse GKRP:P446 > L phenocopies the human GKRP:P446 > L substitution despite the higher affinity of human GKRP for GK [25]. From comparison of GKRP and GK protein levels in human liver homozygous for the rs1260326:P446 > L, with a P446L knock-in mouse [26] we show that the mouse model replicates the lower liver GK and GKRP protein levels in human liver of 446LL genotype. The P446L mouse challenged with a high-fat high-sugar diet manifested lower blood glucose and insulin and raised blood cholesterol but not raised triglycerides. It thereby replicates some of the metabolic traits associated with the Chromosome-2 locus. Transcriptome analysis of P446L mouse liver and hepatocytes overexpressing GK demonstrates a link between relative GK-to-GKRP protein excess and altered hepatic cholesterol homeostasis*.*

## 2 Materials and methods

### 2.1 Animals and ethics statement

All animal studies were approved by Newcastle Animal Welfare Ethical Review Board (AWERB No-532) and covered by UK Home Office Licence PC1B78F4. They were conducted in compliance with the Institution's ethical (AWERB) and ARRIVE guidelines. Two mouse lines were used: the H-GCKR-DEL1262-EMI-B6N generated at MRC Harwell (Gckr^em1(IMPC)H^ mouse.phenotype.org) referred to here as Gckr^+/−^ which has a 1262 nt deletion in exons ENSMUSE00000487442 and ENSMUSE00000486780 and the GCKR-P446L-EMI-B6N (referred to as here as P446L) generated as described previously [26]. Mice were housed in environmentally controlled conditions at 20 ± 2 °C, with 12 h light/dark cycle in individually ventilated cages, with *ad libitum* access to water and standard rodent diet unless otherwise stated.

### 2.2 Human liver and ethics statement

Liver biopsies (formalin-fixed paraffin-embedded needle biopsy, n = 48 cases; 32 M; 16 F) with confirmed Nonalcoholic fatty liver disease (NAFLD) with simple steatosis or NASH (nonalcoholic steatohepatitis) were accessed via the European NAFLD Registry [27] from the Newcastle upon Tyne Hospitals NHS Trust (n = 36) and University Medical Centre Mainz (n = 12). They were selected based on the *GCKR* rs780094 genotype from previous analysis [27]. Informed consent was obtained to use their surplus tissue. Prospectively recruited patients were over 18 years of age. Collection and use of biological samples, surplus to diagnostic requirement for research was approved by the North East -Tyne & Wear South Research Ethics Committee (Fel:15/NE/0150, IRAS:178,250, UK) and by Ethikkommission der Landesärztekammer Rheinland-Pfalz (Ref: 2018–13269, Germany). Fixed-tissue was genotyped for *GCKR* rs1260326 (ThermoFisher TaqMan, Assay ID C_2862880_1 primers). Histopathology was assessed from Haematoxylin and Eosin (H&E) and Sirius red fast green (SRFG) stained sections by an expert liver pathologist (DT) blinded to genotype and scored for grade of steatosis (0–3), hepatocyte ballooning (0–2), lobular inflammation (0–3) and NAFLD activity score [28]. From this 40 cases (23CC, 17 TT) were used for the study.

### 2.3 P446L mouse model

P446L mice were fed on standard rodent diet unless otherwise indicated. For the high-fat diet (HFD) and the high-fat high-sugar diet (HFHSD) studies which were run consecutively male mice were used and the food pellets (Special Diet Services, Whitham Essex #824018) contained (g% w/w): casein 26.5; choline bitartrate 0.296; l-cystine 0.398; lard 18; rice starch 18.428; cellulose 6.16; soya oil 4.315; sucrose 20.343; mineral mix 4.315; vitamin mix 1.233 and was by energy (kcal): 45% fat (36.3%, lard; 8.7%, soya oil); 20% protein; 35% carbohydrate. For the HFHSD the drinking water contained 10% glucose and 5% fructose. Food intake was monitored weekly. On termination of the studies tissues were harvested after isoflurane anaesthesia and blood was collected from the heart/thoracic cavity. Liver sections from the left lateral lobe were frozen in liquid nitrogen for protein, lipid and RNA analysis and formalin fixation.

### 2.4 Mouse hepatocyte isolation

Hepatocytes were isolated from mice of the GCKR-DEL1262-EMI-B6N (Gckr^−/−^ genotype) or the GCKR-P446L-EMI-B6N (P446L, PP and LL genotypes) strains fed on standard rodent diet, by collagenase perfusion [29]. They were cultured in monolayer in Minimum Essential Medium (MEM) containing 7% v/v neonatal calf serum, 10 nM insulin, 10 nM dexamethasone [29]. After cell attachment (∼2–3 h), the medium was replaced by serum-free MEM containing the adenoviral vectors. For other experiments it was replaced by serum-free MEM containing 10 nM insulin and 10 nM dexamethasone. All experimental studies were performed after overnight culture.

### 2.5 Transfection of human GKRP and mouse GKRP in mouse hepatocytes

Hepatocytes isolated from either *Gckr*^*−/−*^ mice (male, age 8–24 wk) or P446L mice (homs 446LL, 8–24 wk) were used. Male mice were used in all studies except for the hepatocyte RNA-sequencing study (Figure 2) which was on female P446L mice (n = 3, age 14 wk). Hepatocytes from these genotypes do not show nuclear sequestration of endogenous GK. After hepatocyte attachment (2–3 h) the serum-containing MEM was replaced by serum-free MEM containing the GKRP-adenoviral vectors (human *GCKR*(Adv-209743; Ref-seq BC130481: 446P or 446L) and mouse *Gckr* transcript-1 (Ref seq XM_006503881.3: 446P or 446L); *Gckr*-transcript-2 (Adv:259,984; BC012412: 446P or 446L) and *Gckr*-X1 (XM_006503882); *Gckr*-X2 (XM_006503883: 446P) at final titres between 5 × 10^6^ and 40 × 10^6^ pfu/ml (corresponding to MOI 5–50 pfu/cell). For the GK-adenoviral vector [30], a titre determined empirically to increase GK by 2–4 fold above endogenous was used. After incubation with the vectors (4–5 h), the medium was replaced with MEM containing 10 nM dexamethasone and 10 nM insulin for overnight culture.

**Figure 1:**
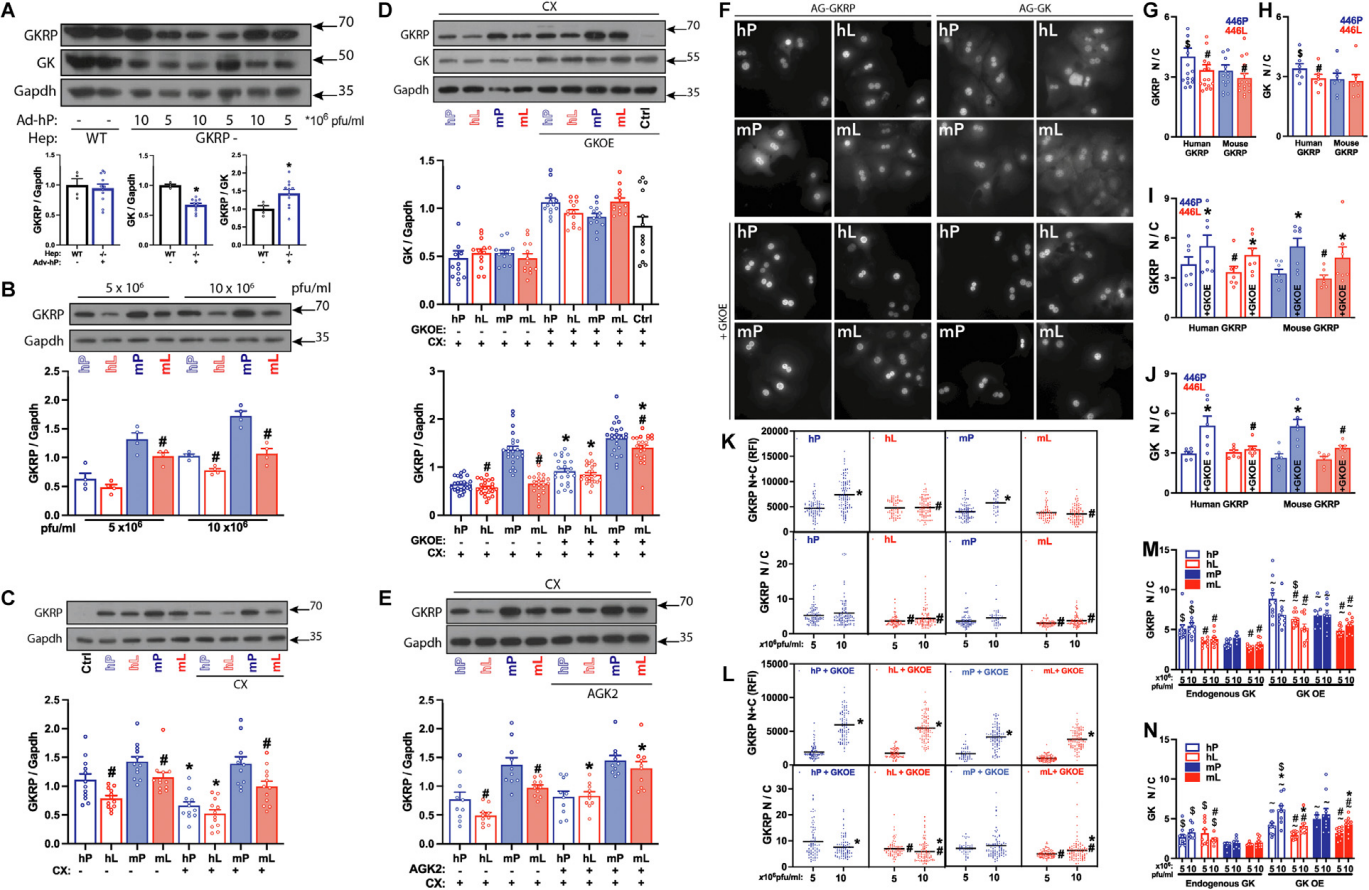
**P446 > L substitution in human or mouse GKRP compromises protein expressivity and GK nuclear sequestration**. Hepatocytes isolated from GKRP-deficient mice were treated with adenoviral vectors (at 5 × 10^6^ or 10 × 10^6^ pfu/ml) to express human or mouse GKRP:446 P/L (hP, hL, mP, mL) and where indicated with a vector to overexpress GK (−/+ GKOE: D,F,I,J,L-N) and cultured for 24–30 h. Inhibitors (CX, AGK2: C-E) were added for the last 6 h culture. A) GKRP-deficient hepatocytes transfected with human GKRP:446 P at 5 × 10^6^ or 10 × 10^6^ have comparable GKRP immunoactivity, lower endogenous GK and higher GKRP/GK immunoactivity ratios than wild-type hepatocytes. Means ± SEM, n = 5, *∗P < 0.05*. B) Lower expressivity of GKRP:446 L variant (hL, mL vs hP, mP) in transfections at 5 × 10^6^ or 10 × 10^6^ pfu/ml is more prominent at the higher titre, n = 4, *#P < 0.05* 446 L vs 446 P. C) Lower protein stability of human compared with mouse GKRP after incubation with cycloheximide (CX). Pooled data of transfections with 5 × 10^6^ and 10 × 10^6^ pfu/ml and cultured for 24 h followed by 6 h culture −/+ 10 μM CX, n = 5, ∗*P < 0.05* effect of CX; *#P < 0.05*, 446 L *vs* 446 P. D) GK overexpression by ∼2-fold relative to endogenous GK promotes stabilization of human GKRP:446 P/L and mouse GKRP:446 L. Means ± SEM, n = 5 hepatocyte preparations; pooled data of 5 × 10^6^ and 10 × 10^6^ pfu/ml transfections. *∗P < 0.05* effect of GKOE; *#P < 0.05,* 446 L vs 446 P. E) Stabilization of GKRP:446 L by a Sirt2 inhibitor (10 μM AGK2) in transfections at 5 × 10^6^ pfu/ml. Means ± SEM, n = 3, *∗P < 0.05* effect of AGK2; *#P < 0.05,* 446 L vs 446 P. F) GKRP and GK immunostaining in transfections with human (hP, hL) or mouse (mP, mL) GKRP at 10 × 10^6^ pfu/ml at endogenous GK (upper panels) or with GK overexpression (+GKOE). G) Lower nuclear sequestration (N/C, nuclear/cytoplasmic ratio) of GKRP:446 L in hepatocytes expressing endogenous GK, n = 15–16 hepatocyte experiments. H) Greater nuclear sequestration of endogenous GK with human compared with mouse 446 P. n = 7; *#P < 0.05,* 446 L vs 446 P; *$P < 0.05* human vs mouse (G,H). I) Nuclear sequestration of GKRP:446 P or 446 L is increased by GKOE, n = 7. J) Nuclear sequestration of GK during GKOE is lower with GKRP:446 L, n = 6; *#P < 0.05,* 446 L vs 446 P; ∗P < 0.05 effect of GKOE (I,J). K-M) Comparison of GKRP expressivity (N + C intensity) and nuclear GKRP sequestration (N/C ratio), in a representative experiment (−/+ GKOE), with data points representing individual hepatocytes (K,L) or mean value of fields (M,N). $ P < 0.05 human vs mouse; *#P < 0.05,* 446 L vs 446 P; ∗*P < 0.05,* 10 × 10^6^ vs 5 × 10^6^ pfu/ml; ∼*P < 0.05* effect of GKOE (M,N). K) At endogenous GK, with GKRP expressed at 5 × 10^6^ or 10 × 10^6^ pfu/ml, expressivity of GKRP:446 L (N + C), was compromised at 10 × 10^6^ pfu/ml, but nuclear sequestration (N/C) of 446 L was lower at both 5 × 10^6^ or 10 × 10^6^ pfu/ml. L) With GKOE, GKRP:446 L expressivity was not compromised at 10 × 10^6^ pfu/ml and GKRP:446 L N/C was lower at both titres. M) GKRP N/C summary for (K)–(L): showing greater N/C for human vs mouse GKRP ($) and lower N/C for 446 L (#). N) GK N/C summary showing greater N/C for human vs mouse and lower N/C for 446 L vs 446 P (#) with GKOE.

**Figure 2:**
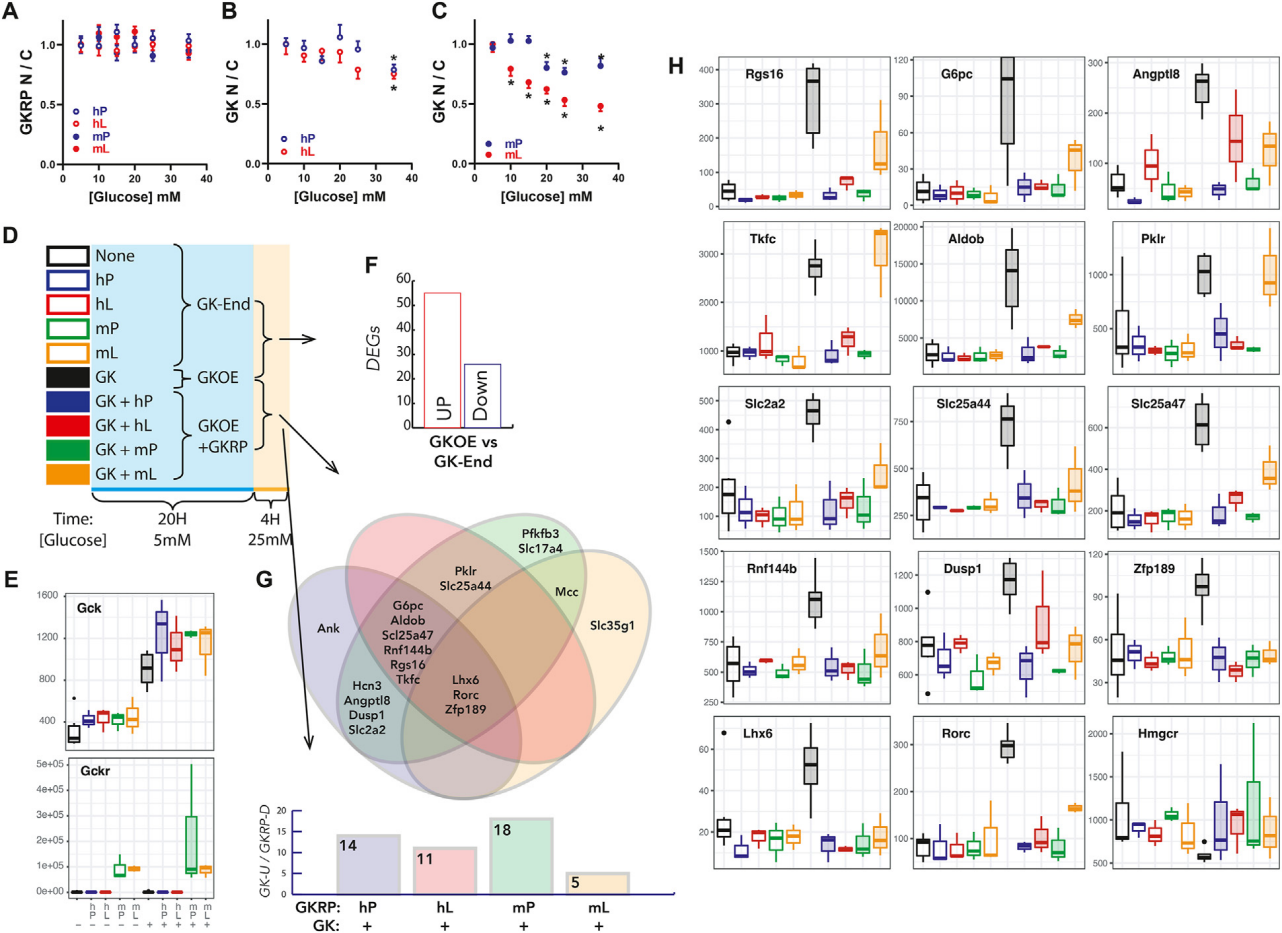
**Functional assessment of human and mouse GKRP:P446 > L by glucose-dependent translocation and transcriptome analysis**. A-C) Effects of glucose concentration (5–35 mM) on nuclear sequestration (N/C: nuclear/cytoplasmic ratio) of GKRP (A) and GK (B),(C) in hepatocytes from GKRP-deficient mice transfected (10 × 10^6^ pfu/ml) with human or mouse GKRP:446 P/L (hP, hL, mP, mL) and cultured for 24 h. A) Nuclear sequestration of GKRP is independent of glucose concentration. B) Translocation of GK at 35 mM glucose in hepatocytes expressing human GKRP (hP or hL). C) Translocation of GK at ≥20 mM glucose and ≥10 mM glucose in hepatocytes expressing mouse GKRP mP or mL, respectively, n = 3 hepatocyte preparations, ∗*P* < 0.05 relative to 5 mM glucose. D-H) Gene expression by unbiased RNA-transcriptome analysis in hepatocytes that were either untreated (none) or transfected (10 × 10^6^ pfu/ml) with human (hP, hL) or mouse (mP, mL) GKRP and either without (GK-End) or with GK overexpression (GKOE) and cultured for 24 h followed by 4 h incubation with 25 mM glucose before RNA extraction and processing for RNA-Sequencing, n = 3 hepatocyte preparations (D). E) Gene counts for mouse *Gck* and *Gckr* showing expression by the adenoviral vectors. F) Numbers of significantly up-regulated or down-regulated genes by GKOE relative to the 5 combined groups of GK-End (none, hP, hL, mP, mL). G) Venn diagram showing genes down-regulated by GKRP with GKOE relative to GKOE alone. H) Gene counts for 14 of the genes up-regulated by GK and down-regulated by GKRP and for *Hmgcr* which is conversely regulated by GK and GKRP showing heterogeneity of response (hP, mP, hL, mL).

### 2.6 Hepatocyte incubations for glucose metabolism and cell metabolite analysis

Glucose phosphorylation and glycolysis in hepatocytes were determined after 24 h culture by incubation with [2–^3^H]glucose or [3-^3^H]glucose, respectively as described [17]. Cell ATP was determined by a Luciferase coupled luminometric assay (Sigma FLAA) and glucose 6-P and glycerol 3-P were determined by fluorimetric assays [29].

### 2.7 Immunostaining for GKRP and GK in hepatocyte monolayers

Hepatocyte monolayers were fixed for 30 min in 4% (w/v) paraformaldehyde in PBS, treated with sodium borohydride (1 mg/ml in PBS, 10min) followed by Triton X100 (0.2% w/v) in PBS (10 min) and stained separately for GKRP (SC-6340, *GCKR*-N19, 4 μg/ml) or GK (SC-7908, GCK-H88 at 4 μg/ml or PT15629 at 1:200 dilution) with Triton-X100 (0.1% w/v), BSA (1%) in PBS (2 h). For GKRP immunostaining the secondary antibody was Alexa-Fluor-488 anti-goat-IgG and for GK Alexa-Fluor-546 anti-rabbit-IgG (1:50 dilution) with 1% Triton-X100, BSA (1%) in PBS (1 h). Nuclei were counterstained with DAPI. Cells were imaged using a Nikon E400 microscope (60X). For each coverslip 5 fields were selected based on DAPI staining representing 6–20 nuclei/field. The mean pixel intensity for the nucleus and cytoplasm was analysed using ImageJ and the nuclear-to-cytoplasmic (N/C) ratio was calculated for each coverslip from the 5 fields averaging 40–50 nuclei. With the exception of the data in Figure 1K–N which represents a single hepatocyte preparation (representative of 2) other data (Figure 1G–J) represent individual hepatocyte experiments.

### 2.8 GK activity in mouse liver and hepatocyte monolayers

Liver samples (40–80 mg) were homogenized in 15 volumes of 100 mM KCl, 50 mM HEPES, 1 mM EDTA, 2.5 mM DTT, pH 7.5 and centrifuged at 100,000 g for 45min [31]. The microsomal pellet and floating lipid layer were discarded and assays for low-K_m_ hexokinase and total hexokinase activity were performed on 5 μl of 100,000 g supernatant in an assay containing 100 mM KCl, 50 mM HEPES, 2 mM MgCl_2_, 5 mM ATP-Mg^2+^, 0.5 mM NAD, 2.5 mM DTT, 8U/ml glucose 6-phosphate dehydrogenase (Sigma, G8404), pH 7.8 and 0.5 mM or 100 mM glucose (340 nm, Spectramax, Molecular Devices). GK activity (Units/g, representing μmol/min per g) was determined by subtraction of low-K_m_ hexokinase [31]. Hepatocytes were extracted and assayed as in [16,17].

### 2.9 GKRP and GK immunostaining in human and mouse liver

Human liver and P446L mouse liver were immunostained using the Ventana automated system (Roche, BenchMark XT) with the standard retrieval protocol. For GKRP immunostaining a goat antibody (SC-6340 at 1:800 dilution) with anti-goat-HRP OMNIMAP (Roche, 6607233001) and for GK immunostaining either of two rabbit polyclonal antibodies (SC-7908, 1:100; PT-159629, 1:800) were used with anti-rabbit-HRP OMNIMAP (Roche, 5269679001). HMGCR immunostaining was with a rabbit antibody (PT 13533-1-AP, 1:500). Negative controls were with goat IgG (Sigma, 5256) or rabbit IgG (Sigma, I5006).

The slides were scanned x20 magnification with Aperio Image Software (Leica Biosystems). The scans were annotated for stained regions of interest. Quantitative image analysis of the annotated regions was performed using Aperio Brightfield Image Analysis Toolbox Software (Leica Biosystems) for nuclear H-scores and cytoplasmic H-scores (GKRP and GK) or cytoplasmic H-scores (HMGCR). For GKRP and GK the Aperio Software default thresholds were used for nuclear staining (200, 160, 120 pixel intensity) and cytoplasmic staining (210, 180, 150 pixel intensity). For HMGCR the cytoplasmic thresholds were set at 235, 230, 225. The data was exported into MicroSoft Excel for further analysis. The quantitative analysis data exported comprised the total numbers of hepatocyte nuclei and surrounding cytoplasmic regions within the annotated regions and the percentages corresponding to the 3 intensity thresholds. Nuclear and cytoplasmic H-scores were determined according to the formula: ([% weak staining] + [% moderate staining x 2] + [% strong staining x 3]) yielding a range of 0–300.

### 2.10 Histopathology scoring of mouse liver

Mouse liver sections were stained with H&E and SFRG. Histopathology was assessed for steatosis, lobular inflammation, hepatocyte ballooning and fibrosis according to the NASH score [28] by an expert liver pathologist (DT), blinded to genotype. Other histological features including portal inflammation and lipogranulomas were recorded. Semi-quantitative evaluation of sinusoidal fibrosis was based on a 3-tiered system (0–2) where 0 is none, 1 is focal (zone 3), and 2 is extensive (zone 3 and beyond).

### 2.11 Western blot GKRP and GK analysis in mouse liver and hepatocytes

Frozen liver or mouse hepatocytes were lysed in 100 mM NaCl, 25 mM NaF, 2 mM EDTA, 0.1 mM Na_3_VO_4_, 100 mM Tris–HCl pH 7.4, 0.1% Triton X-100, 1 mM benzamidine, protease inhibitors (Sigma P8340), sonicated (MSE-Soniprep 150), centrifuged (14,000 g, 10 min) and supernatant protein determined (Bio-Rad #5000006). Proteins (10–40 μg) were resolved by SDS-PAGE on 10% SDS or 4–12% SDS(Bio-Rad 456-8093) gels, electrotransferred to PVDF membrane and immunoblotted (GK, Proteintech PT15629; GKRP, rabbit-AZ680) followed by horse radish peroxidase conjugated anti-rabbit IgG (1:5000 in 5% BSA/TBST) and developed with Enhanced Chemiluminescence (Perkin Elmer) and exposed to X-ray film. Band densitometry was determined using ImageJ.

### 2.12 RNA extraction and RT-qPCR

Frozen liver or mouse hepatocytes were extracted in Trizol and the RNA was DNase treated (Sigma, 04716728001). cDNA was synthesized from total RNA (1 μg) using MMLV (Promega M1705) and amplified using GoTaq Probe qPCR Master Mix (Promega A6002) with the primers indicated (Supplementary Table-1). Relative gene expression was determined using the delta–delta Cycle threshold. For liver, transcripts were normalized to *RplpO* and for mouse hepatocytes (human *GCKR* and mouse *Gckr*) to *Gapdh*.

### 2.13 RNA extraction and RNA-sequencing

RNA was extracted from mouse livers (n = 74) and hepatocyte cultures (n = 36) using Trizol and purified using RNeasy kit (Qiagen #74104) with on-column DNase digestion (Qiagen #79254). Samples were submitted to the Newcastle University Genomics Core for quality assessment and samples with a RIN value > 6 were used for sequencing. RNA-Seq sample quality was assessed via FastQC and visualized with MultiQC. All samples had quality scores >35, no adapter contamination, and therefore none required trimming. Transcript-level counts were obtained using Salmon run against the mouse reference transcript sequences (release M26, GRCm39) from Gencode (gencodegenes.org/mouse/). Differential gene expression was determined using the DESeq2 package in R via RStudio. Differentially expressed genes (DEGs) were defined as an adjusted P-value (FDR) <0.05 combined with a fold-change increase or decrease of 30%. One mouse, wild-type #7 (of 10), was removed from all analyses following a heatmap showing that its gene expression differed from all other mice. Pair-wise comparison of the different genotypes were carried out to determine gene expression changes.

### 2.14 Blood and tissue analysis

Blood glucose was determined by tail vein sampling using a glucose meter (Roche, Accu-chek) and plasma insulin by ELISA (Mercodia, #10-1247-01). For oral glucose tolerance tests mice were fasted for 2 h, gavaged with glucose (2 g/kg body wt) and tail vein blood sampled at time 0 (before gavage) and intervals indicated. Glucose area under the curve (AUC) was corrected for baseline at time zero. For insulin tolerance tests mice were fasted for 5 h and injected intraperitoneally with insulin (Actrapid, 1Unit/kg body wt) and tail vein blood sampled for glucose at time 0 (before insulin) and intervals indicated. For lipid analysis, blood was collected into heparinized tubes and sedimented 10,000 g for 10 min. Plasma triglycerides (GPO-POD), total cholesterol (CHO-POD), LDL-cholesterol (CHO-PAP), HDL cholesterol, inorganic phosphate (Molybdate) were determined on the AU680 Analyzer at Harwell, Mary Lyon Centre Pathology. Liver lipids were extracted in chloroform/methanol (1:2) and the lipid extract in the chloroform fraction was dried was reconstituted in isopropanol. Liver triglyceride was assayed with WAKO-triglyceride kit (Alpha Laboratories, 290–63701) and cholesterol with a Cholesterol Kit (Cambridge BioSciences, CAY10007640).

### 2.15 Statistical analysis

Statistical analysis was completed using Prism 8 Software (GraphPad Software Inc) or SPSS (v27). Data is shown as mean ± standard error of the mean, except for the gene counts for RNA-seq (which are median and range) or associations and histopathology which are categorical scores. Statistical comparisons between groups of continuous variables was assessed by the two-tail t-test for parametric data and for histopathology by Chi-square test for categorical variables. Statistical significance is set at 0.05 unless otherwise stated.

## 3 Results

### 3.1 Expression of human or mouse GKRP (446P or 446L) in GKRP-deficient mouse hepatocytes

#### 3.1.1 Nuclear GKRP sequestration for mouse *Gckr*-transcript-1 but not *Gckr*-transcript-2

**Figure S1:**
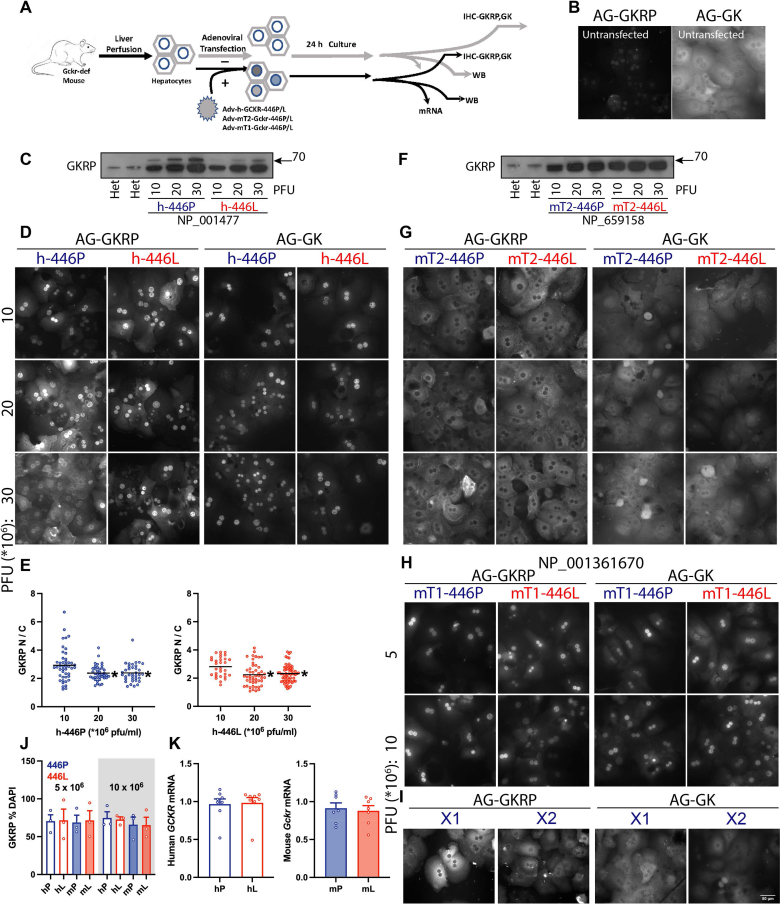
**Optimization of adenoviral vector titres for comparing human and mouse GKRP**. A) Experimental design: hepatocytes isolated from GKRP-deficient mice were cultured in monolayer and transfected with adenoviral vectors for expression of human or mouse GKRP(446P or 446L) at titres of 5–30 × 10^6^ pfu/ml and cultured for 24h for analysis of mRNA, immunoactivity and immunostaining for GKRP (AG-GKRP) and GK (AG-GK). B) No nuclear GKRP or GK immunostaining in un-transfected GKRP deficient hepatocytes. C-E) Nuclear GKRP and GK staining in transfections with human GKRP (NM_001486.4; NP_001477.2) h-446 P/L at titres of 10,20,30 × 10^6^ pfu/ml (C) GKRP immunoreactivity compared with untransfected GKRP^+/−^ hepatocytes (Het). (D) Representative images of nuclear staining of GKRP and GK. E) Maximum nuclear sequestration of GKRP at the lowest titres of 10 × 10^6^ pfu/ml, measured from the nuclear / cytoplasmic (N/C) intensity ratio, ∗P < 0.02 vs 10 × 10^6^ pfu/ml. F-G) Exclusive cytoplasmic staining for GKRP and GK in hepatocytes transfected mouse Gckr-Transcript-2 (NM_144909.2; NP_659158.1) 446 P/L. (F) GKRP immunoreactivity (mouse transcript-2) compared with untransfected GKRP^+/−^ hepatocytes (Het). (G) Immunostaining showing exclusive cytoplasmic staining for GKRP and GK. H) Nuclear GKRP and GK staining in transfections with mouse GKRP-Transcript-1 (NM_001374741.1; NP_001361670.1) 446 P/L at 5 × 10^6^ and 10 × 10^6^ pfu/ml. I) Lack of nuclear staining in cells transfected with Gckr-X1 (XM-006503882) and Gckr-X2 (XM-006503883). J) Similar cellular transfection efficiency at 5 × 10^6^ and 10 × 10^6^ pfu/ml for human GKRP (hP,hL) or mouse T1 (mP,mL) determined from GKRP stained nuclei % DAPI staining, n = 3 hepatocyte preparations. K) Similar Human *GCKR* or mouse *Gckr*-T1 transcript levels (normalized to Gapdh mRNA) for 446P and 446L vectors in transfections with human or mouse GKRP:446P or L (at 5 or 10 × 10^6^ pfu/ml). Means ± SEM for n = 8 (human GKRP), n = 7 (mouse GKRP) hepatocyte experiments.

**Figure S2:**
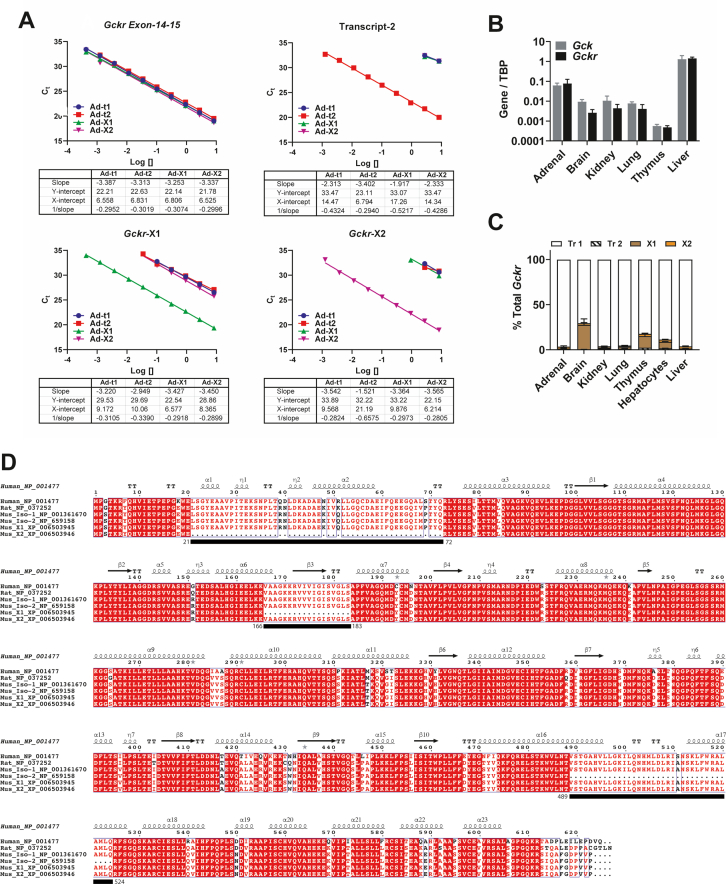
***Gckr* transcript-1 is the predominant transcript expressed in mouse liver and mouse primary hepatocytes**. A-C) Relative abundance of *Gckr* transcripts-1,2, X1 and X2 in mouse tissues determined using primer sets targeting either a common sequence (spanning exons 14–15) or spanning the deletion sites of transcript-2, X1 and X2. A) Relative selectivity of the 4 primer sets determined against RNA extracts from cells transfected with adenoviral vectors (10 × 10^6^ pfu/ml) encoding the 4 transcripts: *Gckr*-t1 (RefSeq XM_006503881.3), *Gckr*-t2 (RefSeq BC012412), *Gckr*-X1 (RefSeq XM_006503882) and *Gckr*-X2 (XM_006503883). B) *Gck* and *Gckr* mRNA are expressed at highest levels in liver. C) *Gckr*-transcript-1 is the predominant transcript in liver and hepatocytes. (B–C) Means ± SEM for n = 4–8 extractions. D) Amino acid alignment of mouse GKRP isoforms 1 and 2, predicted isoforms X1 and X2 versus human and rat sequences using Clustal Omega and visualized via Esprit3(Robert, X. and Gouet, P. (2014) “Deciphering key features in protein structures with the new ENDscript server”. Nucl. Acids Res. 42(W1), W320–W324 - https://doi.org/10.1093/nar/gku316). Showing deleted regions of Isoform 2, X1, and X2.

We used adenoviral vectors to express human or mouse GKRP:446 P or 446 L in hepatocytes isolated from GKRP-deficient mice (Figure S1A). In hepatocytes not transfected with vectors there was no detectable nuclear staining for GKRP or GK (Figure S1B). Transfection with human GKRP:446 P or 446 L at adenoviral titres of 10–30 × 10^6^ pfu/ml resulted in intense nuclear staining for GKRP and the endogenous GK accumulated in the nucleus (Figures. S1C and D). The GKRP nuclear/cytoplasmic (N/C) intensity ratio was greatest at the lowest titre of 10 × 10^6^ pfu/ml (Figure. S1E), indicating accumulation of GKRP in the cytoplasm at higher titres. Transfection with vectors for mouse GKRP-T2 (NM_144909.2), previously designated canonical [32] showed exclusively cytoplasmic GKRP and GK staining (Figures. S1F and G). Transfection with GKRP-T1 (NM_001374741.1, previously designated *Gckr*-X1, which shares 87.5% identity with human GKRP, showed nuclear staining for GKRP and GK similar to human GKRP (Figure 1H vs 1D). Mouse GKRP-T2, which has a 108bp deletion in exon-17, and two other mouse *Gckr* transcripts: XM-006503882; XM-006503883, now designated X1, X2 also encode exclusively cytoplasmic proteins (Figure. S1I). Using primers spanning deletions of T2, X1, X2 we confirmed that *Gckr*-T1 is the predominant transcript and that *Gckr*-T2 is not expressed in mouse liver (Figures. S2A–C). Amino acid alignment of human GKRP, rat GKRP and mouse GKRP isoforms encoded by Gckr-T1 (Isoform-1); Gckr-T2 (Isoform-2), Gckr-X1 (X1) and Gckr-X2 (X2), shows that Isoform-2 expressed in [32] encodes a protein missing residues 489–524, whereas X1, skipping exon-7 is missing residues 166 to 183 and X2, skipping exon-2 is missing residues 21 to 72 (Figure. S2D). It is noteworthy that only Isoform-1 localizes to the nucleus in hepatocytes (Figure. S1).

GKRP-T1 (Isoform-1) was used in the rest of this study. We next used adenoviral titres of 5 × 10^6^ and 10 × 10^6^ pfu/ml, which have similar cellular transfection efficiency (Figure S1J) for comparison of human GKRP and mouse GKRP-T1 (446 P/L) and data from these 2 titres is either pooled (Figure-1A) or presented separately (Figure-1B). *Gckr* mRNA expression determined by RT-qPCR using primers for mouse or human *GCKR* confirmed equal expression at mRNA level for GKRP:446 P compared with 446 L (Figure. S1K).

In GKRP-deficient hepatocytes transfected with human GKRP:446 P, the GKRP immunoreactivity for the pooled data of 5 × 10^6^ and 10 × 10^6^ pfu/ml titres was comparable to GKRP immunoactivity in wild-type mouse hepatocytes (Figure 1A). However, the endogenous GK protein level was lower than in wild-type hepatocytes, as found previously for other GKRP-deficient mouse models [33,34]. Consequently, the GKRP-to-GK ratio was higher than in wild-type hepatocytes (Figure 1A). In the rest of the study GK was expressed by ∼2–3 fold above endogenous where indicated to attain GKRP-to-GK ratios spanning the physiological range.

#### 3.1.2 Lower expressivity of GKRP:446L protein and the stabilizing effect of GK

Comparison of GKRP immunoreactivity in transfections at 5 × 10^6^ or 10 × 10^6^ pfu/ml showed lower protein levels for 446L compared with 446P, for both mouse and human GKRP, at the higher titre (10 × 10^6^ pfu/ml), with smaller differences at the lower titre (Figure 1B), despite similar mRNA expression for 446L (Figure. S1K). To test for differences in protein stability, the protein synthesis inhibitor cycloheximide (CX), was added for the last 6 h (Figure 1C). There was a greater effect of cycloheximide on human GKRP (446P and 446L), indicating lower stability than for mouse GKRP and the lower immunoactivity of mouse 446 L was modestly accentuated (+CX 29% vs no CX 18%).

We next tested the effect of GK overexpression by ≥ 2-fold (Figure 1D). This showed a stabilizing effect of GK on human GKRP (446P and 446L) and on mouse GKRP:446L immunoactivity and the lower expressivity of both 446 L variants was attenuated.

**Figure S3:**
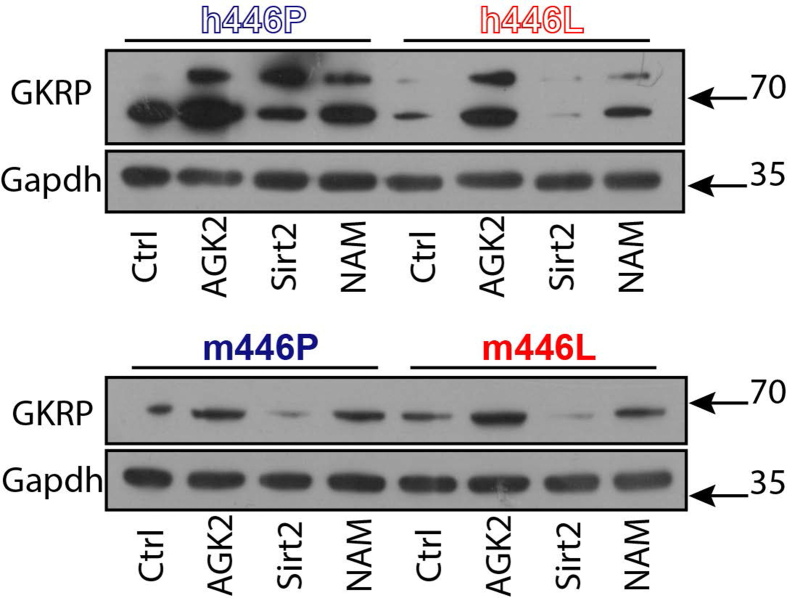
**Increased GKRP immunoreactivity in COS-1 cells treated with Sirt-2 inhibitors**. COS-1 cells were treated with the adenoviral vectors indicated (10^10^ pfu/ml) for expression of human (h) or mouse (m) GKRP (446P or 446L) and where indicated with an adenoviral vector for expression of human SIRT2 (Vector Biolabs, #1519). After 24h culture they were incubated for 6h with 10 μM cycloheximide and the Sirt2 inhibitors, AGK2 (10 μM) and nicotinamide (2 mM, NAM).

A putative role for covalent modification of GKRP by P300 acetyltransferase and Sirt2 deacetylase on GKRP protein stability and interaction with GK has been reported [35,36]. In COS-1 cells transfected with the GKRP-adenoviral vectors, GKRP immunoactivity was increased with the Sirt2 inhibitors, AGK2 and nicotinamide (NAM), and decreased by SIRT2 overexpression (Figure. S3) consistent with a putative stabilizing role for acetylation [36]. When AGK2 was tested in hepatocytes transfected with the GKRP-vectors at 5 × 10^6^ pfu/ml, it increased GKRP immunoactivity for both human and mouse 446L and attenuated the compromised expressivity of the 446L variant (Figure 1E). Cumulatively, this shows lower expressivity of the 446L variant compared with 446P for both human and mouse GKRP that is greater at higher GKRP-to-GK ratios. The lower 446L expressivity is attenuated by GK overexpression and by Sirt2 inhibition consistent with a stabilizing effect of the interaction with GK.

#### 3.1.3 Nuclear sequestration of both GKRP:446P and 446L is dependent on the GK protein level

In GKRP-deficient hepatocytes transfected with adenoviral vectors for human or mouse GKRP at 5 × 10^6^ or 10 × 10^6^ pfu/ml there was large intercellular heterogeneity in the nuclear/cytoplasmic (N/C) staining intensity for GKRP and GK (Figure 1F), as occurs for endogenous GKRP and GK in hepatocytes [37]. To test for differences in nuclear sequestration of the 446 L variant, the N/C mean pixel intensity ratio was determined from 60 to 100 nuclei for each transfection condition in each hepatocyte experiment. The data for experiments with only endogenous GK is summarized in Figure 1G,H. Nuclear sequestration of GKRP:446 P was higher (18%) for human GKRP compared with mouse GKRP and it was lower for the 446 L variant by 17% for human and by 11% for mouse GKRP (Figure 1G) with similar but smaller trends for endogenous GK (Figure 1H).

**Figure S4:**
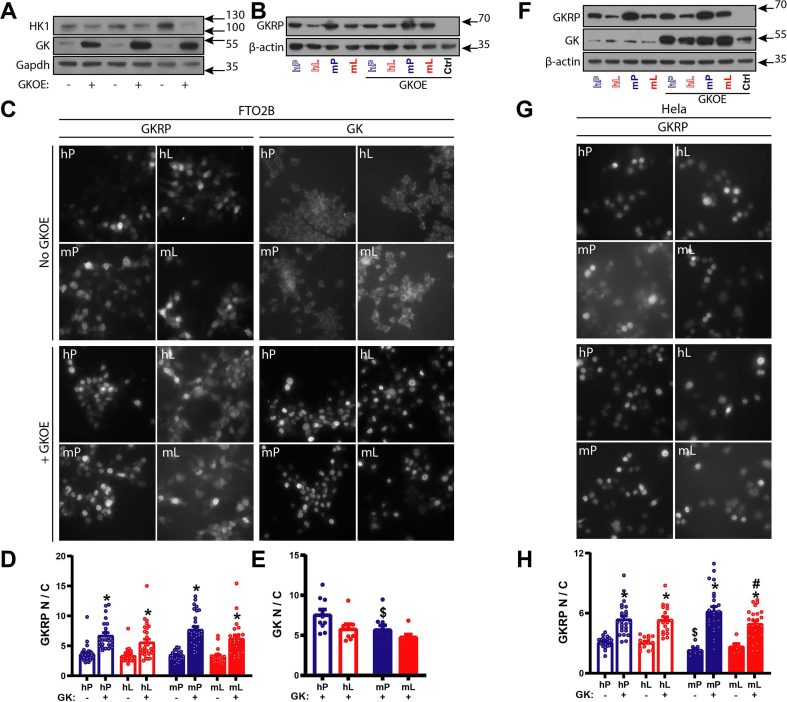
**GK enhances nuclear sequestration of GKRP(446P and 446L) in FTO2B (A**–**E) and HeLa (F–H) cells.** A) GK and hexokinase I immunoactivity in FTO2B cells, untreated or treated with a GK adenoviral vector. B) GKRP immunoactivity in FTO2B cells transfected for expression of human or mouse GKRP (hp, hL, mP, mL at 25 × 10^6^ pfu/ml) and −/+ expression of GK. C) GKRP and GK immunostaining in FTO2B cells. D) Nuclear sequestration of GKRP (N/C intensity ratio) is increased by GK expression *(∗P < 0.001*). Means ± SEM for the fields indicated in 4 experiments. E) GK N/C intensity ratios in GK transfections. *$ P < 0.05* mP vs hP. F-G). GKRP immunoactivity (F) and immunostaining (G) in HeLa cells transfected for expression of human or mouse GKRP (hP, hL, mP, mL) at titres of 10–25 × 10^6^ pfu/ml −/+ expression of GK. H) Nuclear sequestration of GKRP (N/C intensity ratio) is increased by GK expression (*∗P < 0.001*) and is lower with GKRP-446L *(#P < 0.03*) in the presence of GK expression. Means ± SEM for the fields indicated in 2 experiments.

Overexpression of GK increased the sequestration of GKRP in the nucleus for both 446 P and 446 L by 34–37% for human GKRP and by 54–61% for mouse GKRP (Figure 1I), indicating that increased GK protein promotes nuclear GKRP sequestration. When GKRP (446 P or 446 L) was expressed in heterologous cell lines (FTO2B hepatoma and HeLa cells) which lack endogenous GK, and without or with adenoviral-mediated GK expression, the sequestration of GKRP in the nucleus was increased ∼2 fold by GK expression (Figure. S4), confirming a role for GK in sequestering GKRP in the nucleus.

#### 3.1.4 Lower nuclear GK sequestration by GKRP-446L

In hepatocytes overexpressing GK by ∼2-fold above endogenous (Figure 1D), the nuclear accumulation of GK (Figure 1 J) was increased ∼2-fold, if cells expressed GKRP-446 P (human 1.7 ± 0.17, mouse 1.98 ± 0.23 fold,∗P < 0.02), but negligibly for GKRP-446 L (human 1.04 ± 0.10, mouse 1.31 ± 0.16) and nuclear GK accumulation was significantly lower (32–35%, #P < 0.03) with GKRP-446 L indicating a lower affinity for GK of human and mouse GKRP:446 L.

#### 3.1.5 Compromised nuclear GKRP-446L sequestration-independent of lower expressivity

To test whether the lower nuclear sequestration of GKRP-446L (Figure 1G,I) is explained by its lower expressivity (Figure 1B), we compared nuclear sequestration (N/C) and cellular expression (N + C) by immunofluorescent staining at adenoviral titres of 5 × 10^6^ or 10 × 10^6^ pfu/ml (Figure 1K,L). In hepatocytes expressing only endogenous GK (Figure 1K), total cell intensity of the 446L variant was comparable to 446P at the lower titre (5 × 10^6^ pfu/ml) but not at the higher titre (Figure 1K upper panel). However, the N/C ratio was lower for 446 L at both titres (Figure 1K, lower panel), indicating that it is in part independent of compromised expressivity. With GK overexpression (Figure 1L) total GKRP cell intensity for 446L was comparable to 446P at both titres (Figure 1L-upper panel), consistent with GK-mediated stabilization and the GKRP N/C ratios were elevated compared with endogenous GK alone (6–10 vs 3–6) and were lower for 446 L. Figure 1M summarizes the GKRP N/C ratios and the corresponding GK N/C ratios are shown in Figure 1N. At endogenous GK, nuclear GK sequestration was greater for human than mouse GKRP at both titres (Figure 1N), consistent with a higher affinity for GK of human GKRP [25].

Cumulatively, the results support the following conclusions: i) nuclear GKRP sequestration is enhanced by a higher GK-to-GKRP ratio (Figure 1I, Figure S4); ii) human GKRP has a higher affinity for GK than mouse GKRP as shown by the greater nuclear sequestration of endogenous GK (Figure 1H); iii) GKRP-446 L has a lower affinity for GK as shown by the greater sequestration of overexpressed GK in the nucleus with 446P (Figure 1J); iv) lower expressivity of GKRP-446L by immunostaining is apparent at higher GKRP-to-GK ratios (Figure 1K) and concurs with the immunoblotting (Figure 1B-D); v) lower sequestration of GKRP:446L in the nucleus is in part independent of the lower expressivity (Figure 1K) and best explained by its lower affinity for GK; vi) differences between the 446L variant and GKRP-446P are qualitatively similar for human and mouse GKRP.

#### 3.1.6 Glucose-dependent GK translocation and rates of glucose phosphorylation

**Figure S5:**
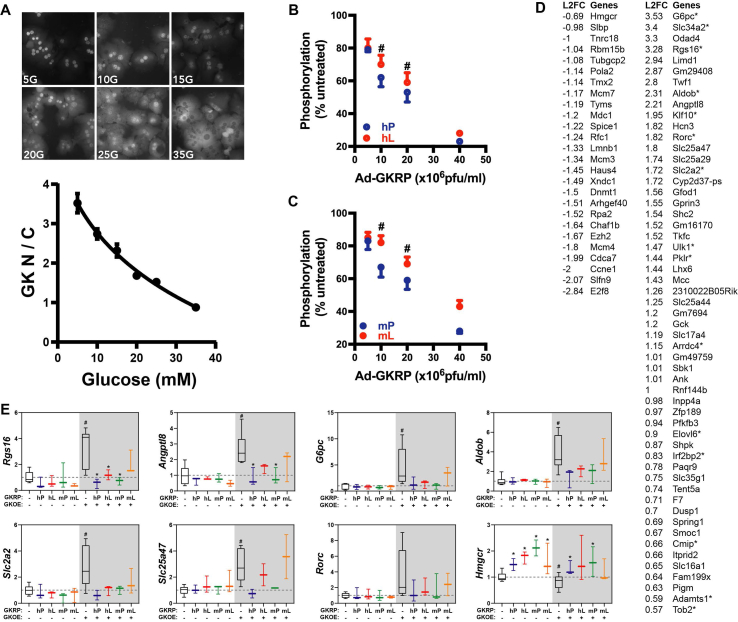
Complementary data to Figure-2: GK translocation; GKRP-inhibition of glucose phosphorylation; Differentially expressed genes by GK overexpression from RNA-sequencing and RT-qPCR validation. A) Translocation of GK by glucose (N/C, nuclear/cytoplasmic intensity) in wild-type 446 PP mouse hepatocytes. Mean ± SEM, 5 fields, 1 hepatocyte preparation. B–C) Inhibition of glucose phosphorylation (metabolism of [2–^3^H]glucose) by human hP, hL (B) or mouse mP, mL (C) GKRP expressed in GKRP-deficient hepatocytes over a range of adenoviral titres 5–40 × 10^6^ pfu/ml showing lower inhibition by hL or mL (#P < 0.05) at 10–20 × 10^6^ pfu/ml. Means ± SEM for 3–12 hepatocyte preparations (B,C). D) List of genes significantly down-regulated or up-regulated (log-2 change) by GKOE relative to endogenous GK (experimental details as in Figure 2 D, n = 3 hepatocyte preparations), ∗denotes genes previously identified as ChREBP target genes by CHIP-sequencing or gene microarrays [[38], [39], [40]]. E) RT-qPCR validation (n = 3 hepatocyte preparations) of 8 genes conversely regulated by GK and GKRP.

Nuclear GK sequestration in rat hepatocytes is glucose-dependent and maximal at 5 mM glucose with half-maximal translocation to the cytoplasm at 10–20 mM glucose depending on the GK-to-GKRP ratio [11,17]. In hepatocytes from wild-type mice glucose-dependence of GK translocation (Figure. S5A) concurred with previous findings in rat hepatocytes. Because nuclear GKRP sequestration is dependent on the GK-to-GKRP ratio (Figure 1I) we tested whether glucose (10–35 mM) affects nuclear GKRP:446 P/L sequestration. There was no significant effect of 10–35 mM glucose on nuclear sequestration of human or mouse GKRP-446 P/L (Figure 2A). However, translocation of GK from the nucleus was induced at 35 mM glucose in hepatocytes expressing human GKRP:446 P or 446 L (22% or 25%, respectively) and at ≥ 20 mM glucose or ≥ 10 mM glucose in hepatocytes expressing mouse GKRP:446 P or 446 L, respectively (Figure 2B–C). Translocation at lower glucose for mouse GKRP, concurs with the higher affinity of human GKRP for GK. Rates of glucose phosphorylation were inhibited by GKRP overexpression with differences between 446 L and 446 P manifesting at adenoviral titres ≥10 × 10^6^ pfu/ml (Figures. S5B and C), indicating that they are in part due to compromised GKRP-446 L expressivity.

#### 3.1.7 Transcriptome analysis of gene counter-regulation by GK excess and by GKRP:P446 > L

To test for functional differences between GKRP:446 P and 446 L we performed RNA-sequencing of hepatocytes expressing human or mouse GKRP (446 P or 446 L) at either endogenous GK or overexpressed GK (10 conditions) after 4 h incubation with 25 mM glucose (Figure 2D,E). GK overexpression caused significant induction of 55 genes and repression of 26 genes (Figure 2F; Figure. S5D) from comparison of GK overexpression alone with the 5 combined groups of endogenous GK (untreated −/+ GKRP-hP,hL,mP,mL). This analysis identified fewer differentially expressed genes (DEGs) than comparison with the untreated group alone but minimizes non-specific genes linked to the adenoviral vector. The genes up-regulated by GK overexpression included candidate ChREBP-target genes [[38], [39], [40]] such as *Pklr, G6pc, Rgs16, Aldob, Klf10, Arrdc4, Slc2a2* and *Rorc* and other genes not previously identified as ChREBP targets such as *Slc16a1*, the lactate and pyruvate transporter and 3 mitochondrial transporters of the Slc25 family including *Slc25a44*, which transports branched-chain amino acids and ubiquinone precursors [41] and *Slc25a47*, which is expressed predominantly in hepatocytes [42]. The genes down-regulated by GK overexpression included *Hmgcr* (Figure. S5D). Eight genes were validated by RT-qPCR (Figure. S5E).

Expression of human or mouse GKRP-446 P/L in cells overexpressing GK, repressed around 60 genes of which 20 were up-regulated by GK overexpression (Figure 2G). The gene counts are shown for 14 of the 20 genes and for *Hmgcr* (Figure 2H). There was heterogeneity of response (446 P vs 446 L) with some genes showing similar repression by 446 P and 446 L (*Lhx6*), others stronger repression by 446 P (*Angptl8, Slc25a47*) or minimal repression by mouse 446 L (*Pklr, Tkfc*). Cumulatively, this identifies ChREBP-target genes and novel genes conversely regulated by GK and GKRP and shows weaker counter-regulation by GKRP-446 L that is more pronounced for mouse GKRP consistent with greater glucose-induced GK translocation (Figure 2C).

### 3.2 Lower GKRP and GK protein in *GCKR* rs1260326-446LL liver and in the P446L mouse

#### 3.2.1 Lower GKRP and GK protein in human liver homozygous for *GCKR* rs1260326-446 L

**Figure 3:**
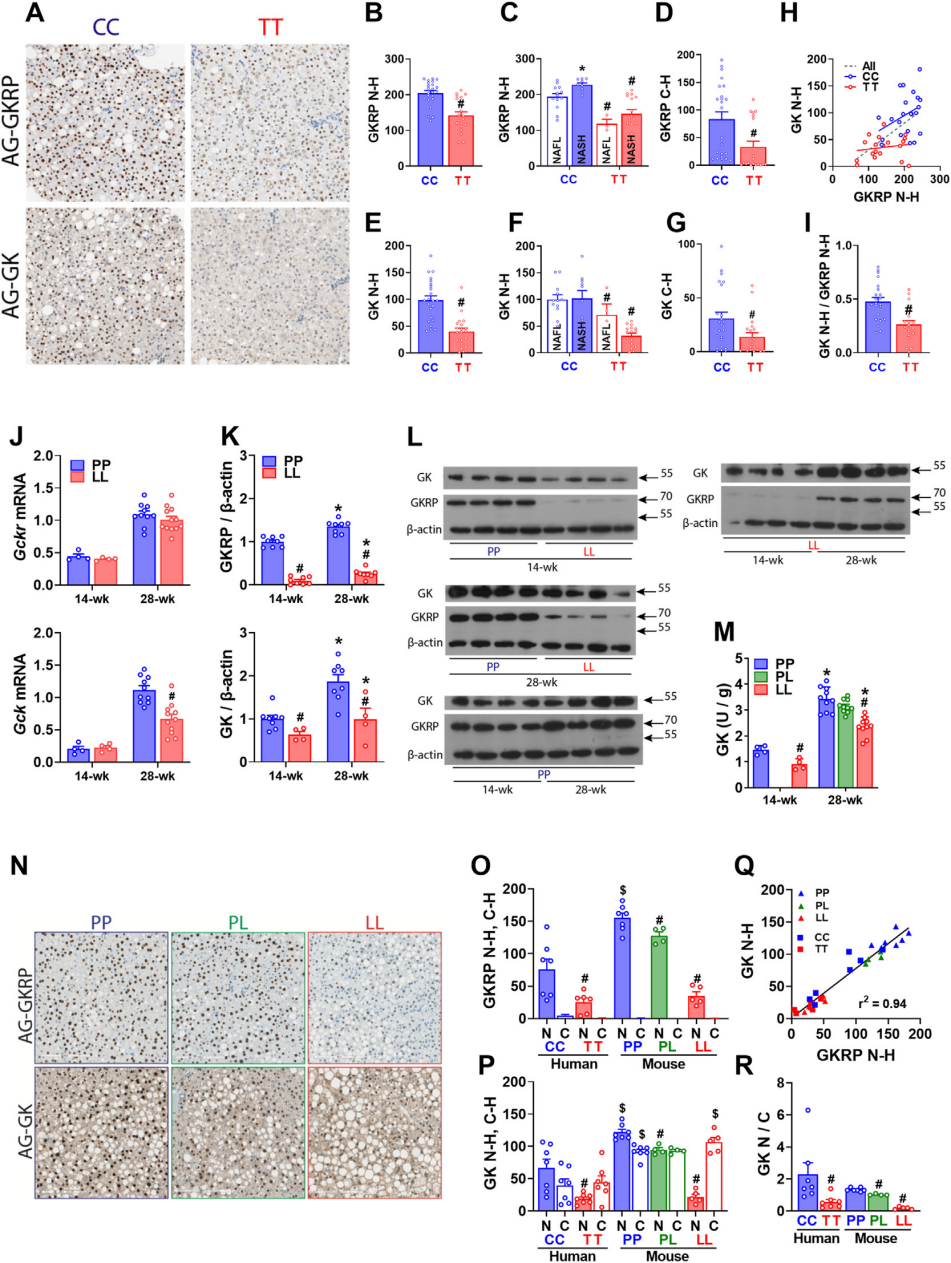
**Lower GKRP and GK protein levels in human NAFLD biopsies of the rs1260326-TT genotype and in the P446L mouse.** A-I) Human NAFLD biopsies genotyped for *GCKR* rs1260326 variant (CC n = 23; TT n = 17) and scored as simple steatosis (NAFL) or NASH were stained for GKRP or GK and nuclear (N–H) and cytoplasmic (C–H) H-scores were determined. (A) representative images. (B–D) Lower GKRP N–H scores by TT genotype for entire cohort (B) or by −/+ NASH diagnosis (C), lower C–H scores (D) (E–G) Lower GK N–H scores by TT genotype for entire cohort (E) or by −/+ NASH diagnosis (F) and lower C–H scores (G) H) Correlation of GK N–H vs GKRP N–H, r^2^ 0.34, *P < 0.0001* for entire data set. I) Lower GK (N–H)/GKRP (N–H) ratio by TT genotype, #P < 0.0003. J-M) GKRP P446L mouse aged 14-wk (regular diet, n = 4,4, PP, LL) or 28-wk (on HFHSD for last 20-wk, n = 10,10, PP, LL): Liver *Gckr* and *Gck* mRNA normalized to *RplpO* (J); GKRP and GK protein (K–L) and GK enzyme activity (M). J) Similar *Gckr* mRNA by genotype but lower *Gck* mRNA in 28-wk mice by LL genotype. K-L) Lower GKRP and GK immunoactive protein (n = 4–8) and lower GK activity (M, n = 4,4,10,10,10) by LL genotype. #P < 0.05, LL vs PP; ∗P < 0.05 28-wk vs 14 wk. N-R) Comparative GKRP and GK immunostaining of human liver biopsies (CC,TT: n = 7,6) and P446L mouse liver (PP, PL, LL: n = 7,4,5) showing representative images for mouse (N) and the N–H and C–H scores. O) Lower GKRP N–H scores for human compared with mouse ($) liver and by TT and LL genotypes (#) and negligible cytoplasmic H-scores. P) Lower GK N–H and C–H for human compared with mouse liver ($) and lower N–H by TT (human) or LL (mouse) genotype (#). Q) Correlation of GK and GKRP N–H scores. R) Relative distribution of GK in nucleus and cytoplasm (N–H/C–H ratio) showing lower nuclear GK sequestration by TT and LL genotypes. *#P < 0.05* by genotype; $*P < 0.05* human *vs*. mouse.

Human liver biopsies from a nonalcoholic fatty liver disease (NAFLD) study [9] that had been genotyped for *GCKR rs780094* (n = 48) were additionally genotyped for *rs1260326*^C > T^, with 23 and 17 confirmed CC and TT, respectively. Of these 11 (CC) and 14 (TT) had a NASH (Nonalcoholic steatohepatitis) diagnosis according to the Clinical Research Network [28]. Immunohistochemical staining for GKRP and GK showed predominant hepatocyte nuclear staining for both genotypes (Figure 3A). Nuclear immunostaining intensity was assessed from the H-score (N–H), a semi-quantitative measure (scale 0 to 300) of the % of cells with high (x3) plus medium (x2) plus weak intensity. For GKRP immunostaining, the TT genotype had lower N–H scores in the entire data set (Figure 3B, *P < 0.00001*), and in steatosis and NASH groups (Figure 3C, *P < 0.004; P < 0.0001*) and the cytoplasmic H-scores (C–H) were also lower for the TT genotype (Figure 3D, *P < 0.001*), showing lower overall GKRP protein by TT genotype. Likewise, for GK immunostaining, the TT genotype had lower N–H scores in the entire group (Figure 3E; P < 0.000001) and in steatosis and NASH groups (Figure 3F) and also lower C–H scores (Figure 3G) indicating lower GK protein by TT genotype. The GK N–H scores for the entire set (CC and TT) correlated with the GKRP N–H scores (Figure 3H, P < 0.001) but with different slopes for the 2 genotypes (CC, 0.40; TT 0.08) and with a significantly higher GK N–H/GKRP N–H ratio for the CC genotype (Figure 3I; CC, 0.47 ± 0.04 vs TT, 0.26 ± 0.04, *P < 0.0003*). This concurs with the lower nuclear GK sequestration in hepatocytes transfected with GKRP-446 L (Figure 1J).

#### 3.2.2 Lower GKRP and GK protein in the GKRP:P446L mouse

Generation of the GKRP:P446L mouse has been reported [26]. At 14-wk of age *Gck*r and *Gck* mRNA levels in LL mice were similar to wild-type (PP) litter mates for male (Figure 3J) and female mice (not shown). However GKRP immunoactivity was very low in LL mice and GK immunoactivity and GK enzyme activity were ∼40% lower (Figure 3K–M). In older mice (28-wk) that were maintained on a high-fat high-sugar diet (HFHSD) for 20 wk, GKRP and GK immunoactivity and GK enzyme activity were higher (∼1.4, 1.7 and 2-fold, respectively 28-wk-PP vs 14-wk-PP), but remained lower by LL genotype (Figure 3K–M).

#### 3.2.3 Comparative GKRP and GK immunostaining in human liver and in the P446L mouse

Concurrent immunostaining of P446L mouse liver (PP, PL, LL, genotypes 28-wk) and human liver (rs1260326: CC,TT, n = 7,7) enabled comparison of GKRP protein levels by species (Figure 3N–R). The mouse GKRP N–H scores were lower in PL and LL genotypes than wild-type (Figure 3O) and the relative values for LL and PP concurred with the GKRP immunoblotting (23% vs 19%, Figure 3J). Cytoplasmic GKRP immunostaining was very low in mouse liver with N–H/C–H ratios >300. For mouse GK immunostaining, the N–H scores of PL and LL genotypes (78% and 18% of PP) showed similar trends as GKRP N–H scores (Figure 3P) and correlated with GKRP N–H scores (Figure 3Q). Cytoplasmic GK immunostaining was high in mouse liver and is estimated at ∼60% of total GK for PP liver based on the GK activity differences by genotype (Figure 3M). Estimates for GK nuclear/cytoplasmic distribution from the N–H/C–H ratio (which is an arbitrary ratio because of lower intensity thresholds for C–H than N–H) showed lower GK nuclear sequestration by LL genotype (Figure 3R).

For human CC livers (446 PP), N–H scores for both GKRP and GK were lower (∼50%) than for mouse PP liver (Figure 3O,P) indicating lower protein levels in human liver. However, nuclear sequestration of GK (N–H/C–H) was similar or higher in human liver (Figure 3R) and similar results were obtained using a different GK antibody (PT15629, not shown). Cumulatively, this shows lower nuclear GK sequestration by 446LL genotype for both human and P446L mouse liver. Additionally, human liver has lower GKRP protein level compared with mouse but similar or higher nuclear GK sequestration consistent with the higher affinity for GK of human GKRP.

### 3.3 Liver transcriptome of the P446L mouse on regular diet

**Figure 4:**
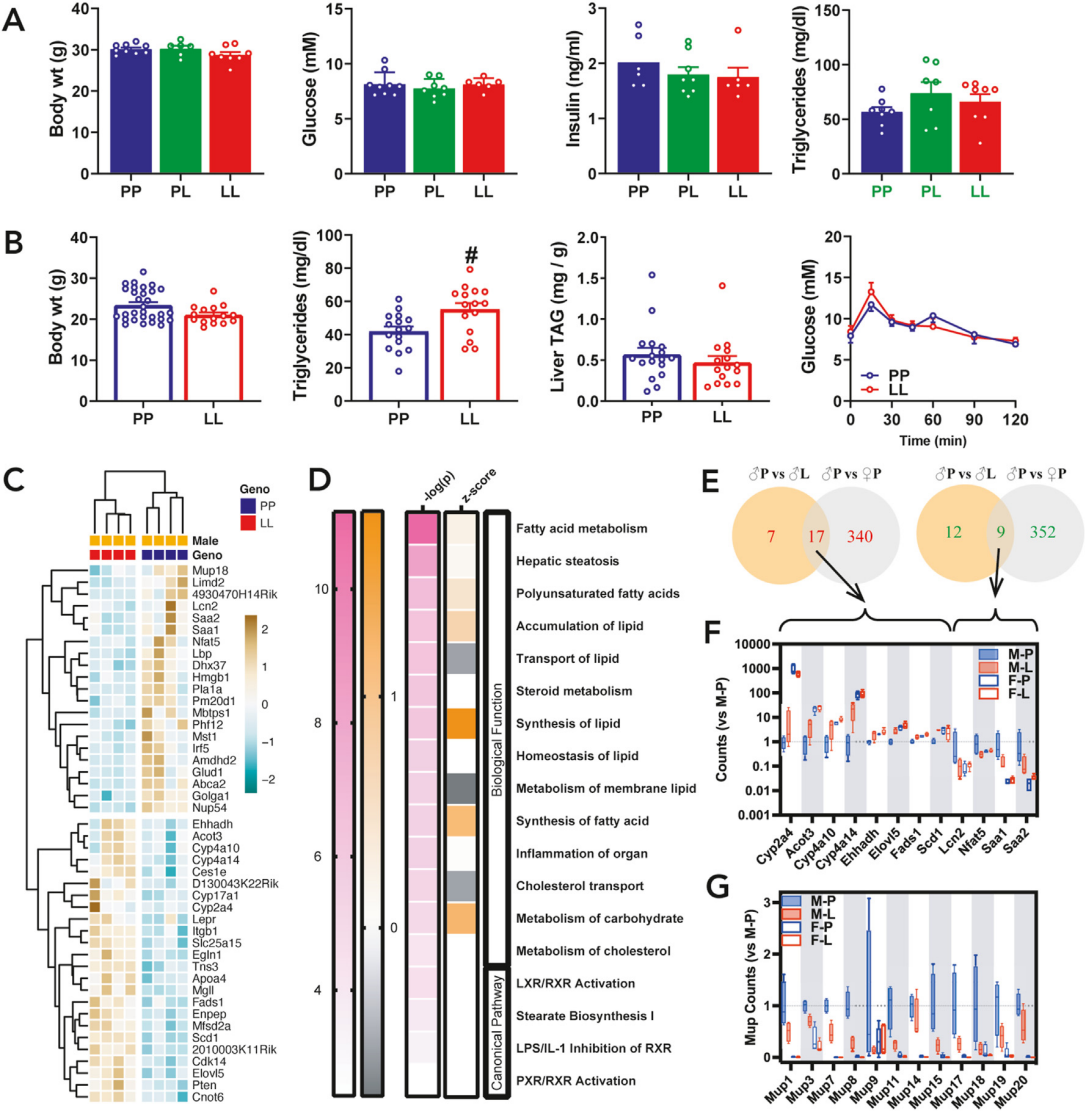
**Blood metabolites and liver transcriptome in the P446L mouse on regular diet**. A) Male mice aged 14 wk: body weight, free-feeding blood glucose, plasma insulin and plasma triglyceride; n = 6–9. B) Female mice aged 14 wk: body weight; plasma triglycerides; liver triglycerides, n = 15–17 and glucose tolerance, n = 7,7. C) Heat map of differentially expressed genes (DEGs, FDR <0.05) in livers of male mice by LL genotype and the direction of the log fold change (LFC) of expression values. D) Top pathways from the Ingenuity Pathway Analysis showing corresponding P value and Z scores. E) Venn diagram showing numbers of common genes that are significantly differentially expressed (upregulation, red; down-regulation green) in male mice by LL genotype and are differentially expressed in female PP vs male PP mice. F) Gene counts for P446L male and female mice for common genes from E). G) Box plot of gene counts from RNA-sequencing for 12 *Mup* genes showing lower expression in male LL mice and in female PP and LL mice; n = 4,4,5,5, (M−P, M-L, F–P, F-L)

GKRP*-*P446L mice on regular rodent diet were fertile with no overt abnormalities. Breeding of PL mice produced PP, PL and LL genotypes in Mendelian ratios. In male mice, despite the lower GKRP and GK protein abundance by LL genotype (Figure 3K), blood glucose, insulin and triglyceride levels were not different (Figure 4A). In female mice, which had higher GK activity than male mice (2.4 ± 0.13 vs 1.5 ± 0.08, U/g, n = 5,4) for the PP genotype, but similar reduced GK activity for the LL genotype (0.9 ± 0.13 vs 0.9 ± 0.13 U/g, n = 5,4) blood triglycerides were higher in LL mice but liver triglycerides and glucose tolerance were not different (Figure 4B).

To assess hepatic adaptations we determined the liver transcriptome of male and female mice (PP and LL, 14-wk) by RNA-sequencing. Unsupervised clustering analysis identified distinct groups by PP and LL genotypes. For the male mice there were 45 differentially expressed genes (DEGs, FDR <0.05) by LL genotype (Figure 4C) and Enrichment Gene Ontology (EnrichGO) and Ingenuity Pathway Analysis (IPA) identified lipid metabolism as the main enriched pathways or processes (Figure 4D). Six of 24 upregulated genes by LL genotype (*Cyp17a1, Ehhadh, Scd1, Enpep, Lepr, Tns3*) corresponded to previously identified ChREBP-target genes in fasted-refed mice [40] or high-glucose challenged hepatocytes or HepG2 [38,39]. In female mice the DEGs by LL genotype did not share common genes with the male DEGs nor did they show significant pathway enrichment by EnrichGO or IPA. Comparison of the male LL DEGs with differential gene expression in female mice showed that several of male-LL, upregulated (17 of 24) and downregulated (9 of 21) genes (Figure 4E) were expressed at higher or lower levels, respectively, in the female mice (Figure 4F), indicating that sexual dimorphic gene expression in part explains the lack of common DEGs.

Comparison of the DEGs by GK or GKRP overexpression from the hepatocyte study (Figure 2) with DEGs by LL genotype in male mice did not identify common genes linked to GK overexpression and identified one gene (*Mup18*) that was up-regulated by GKRP expression in hepatocytes. *Mup18* which was expressed at lower levels in LL male mice (Figure 4C), is one of several major urinary protein (MUP) genes comprising ∼21 coding and ∼30 non-coding genes [43]. MUPs are species-specific ligand-binding 19 kDa proteins of the lipocalin family produced in liver and excreted in urine with roles in communication behaviour and glucose and lipid metabolism [43]. Gene counts for 21 *Mup* genes averaged 43%, 13% and 6% in male-LL, female-PP and female-LL respectively of male PP mice. A sub-set of these *Mup* genes is shown in (Figure 4G).

Cumulatively, the liver transcriptome of male P446L mice on rodent diet identified some ChREBP-target genes and down-regulation of *Mups* but there were no common genes with the GK overexpressed genes identified from the hepatocyte study.

### 3.4 Metabolic adaptations in P446L mouse hepatocytes: attenuated glucose metabolism at elevated glucose but not at 5 mM glucose

**Figure 5:**
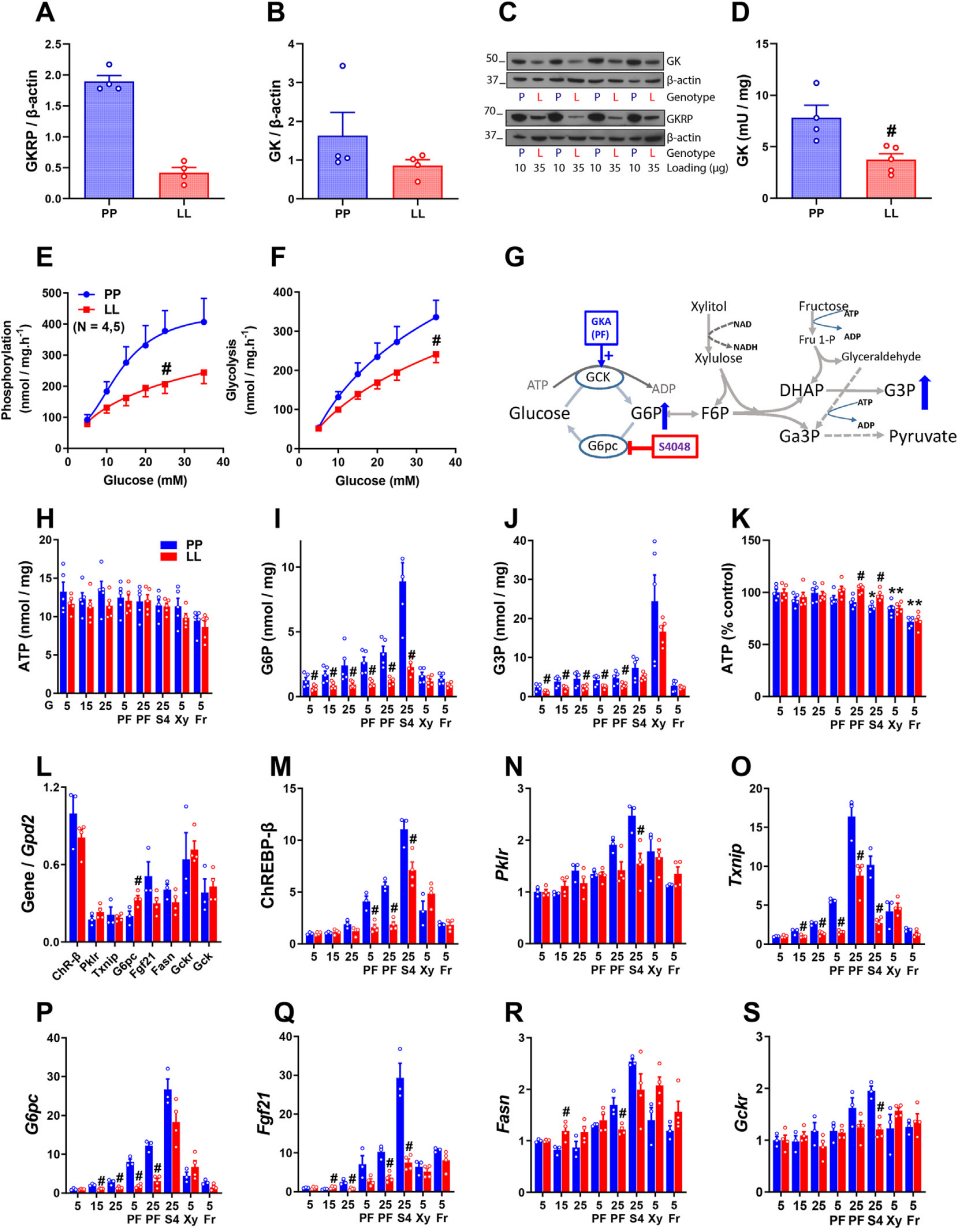
**Functional characterization of glucose metabolism and adaptive response to substrate challenge in hepatocytes from P446L mice**. A-D) GKRP and GK immunoactivity and enzyme activity after 24 h-culture of hepatocytes isolated from male P446L mice (PP and LL genotypes, aged 8–12 wk). (C) Representative immunoblots: protein loading GK 30 μg; GKRP 10 μg (446 PP); 35 μg (446LL); D. Glucokinase activity munits/mg protein (n = 4–5). E-F) Glucose phosphorylation ([2–^3^H]glucose, E) and glycolysis ([2–^3^H]glucose, F) is similar at 5 mM glucose but lower by LL genotype at high glucose, (n = 5,5). G) Metabolic scheme showing metabolism of xylitol and fructose bypass the GK reaction and raises glycerol 3-P (G3P); whereas pharmacological activation of GK with PF-04991532 (PF) or inhibition of glucose 6-phosphatase (G6pc) with S4048 (S4) raises glucose 6-P (G6P). Note that fructose at micromolar concentrations also causes activation of GK via elevation in fructose 1-P and thereby elevation in G6P. However at millimolar concentrations the increase in triose phosphates (G3P) is consequent to flux through ketohexokinase and aldolase B reactions. H–K) Hepatocyte incubations with glucose (15 mM or 25 mM) −/+ the GK activator PF-04991532 (PF, 10 μM) or inhibitor of glucose 6-phosphatase (S4048, 1 μM) or xylitol (Xyl, 2 mM) or fructose (Fru, 10 mM) were for 1 h for determination of cell ATP (H), glucose 6-P (I), glycerol 3-P (J) expressed as nmol/mg protein or ATP normalized to respective 5 mM (K), n = 5,5. L-S) Hepatocyte incubations for RNA extraction and RT-qPCR analysis were for 4 h. (L) Basal mRNA levels of genes indicated normalized to Gpd2 mRNA. (M–S) mRNA levels of the genes indicated normalised to the respective 5 mM glucose control, n = 3,4; #*P < 0.*05 LL vs PP; ∗*P < 0.05* relative 5 mM glucose control.

GK functional activity in hepatocytes is a composite function of the intrinsic amount of GK protein, the molar ratio of GK-to-GKRP, the affinity of GKRP for GK and the intrinsic concentrations of glucose and allosteric effectors of GKRP. To assess functional GK activity in hepatocytes isolated from male P446L mice we compared rates of glucose phosphorylation and glycolysis and metabolite and gene responses to substrate challenge in hepatocytes isolated from 446LL to 446 PP mice after 24 h-culture (Figure 5). The LL hepatocytes had very low (<20%) GKRP protein and ∼50% lower GK protein and total enzyme activity (Figure 5A–D) and thereby a higher GK-to-GKRP molar protein ratio. Rates of glucose phosphorylation or glycolysis were identical in LL and PP hepatocytes at 5 mM glucose but lower in LL hepatocytes at 25–35 mM glucose (Figure 5E,F). During challenge with high glucose (15 mM or 25 mM) −/+ a GK activator (PF-04991532, GKA) or inhibitor of glucose 6-phosphatase (S4048) or with xylitol or fructose (Figure 5G), which bypass the GK reaction [44], basal cell ATP was similar in LL and PP hepatocytes (Figure 5H) but glucose 6-P and glycerol 3-P were higher in PP hepatocytes incubated with high glucose, but not with xylitol or fructose which bypass the GK reaction (Figure 5I,J). Cell ATP normalized to the respective 5 mM glucose controls, was attenuated by xylitol and fructose in both genotypes and by high glucose + S4048 inhibitor only in PP cells, which had higher glucose 6-P (Figure 5K). Basal *Gckr* and *Gck* mRNA levels were similar in LL and PP hepatocytes and likewise mRNA levels for ChREBP-β and several target genes except for *G6pc* which was higher in LL-hepatocytes (Figure 5L). The induction by high glucose challenge of metabolite-responsive genes including ChREBP-β, *Txnip, Pklr, G6pc* and *Fgf21* paralleled the higher glucose 6-P and glycerol 3-P accumulation in the wild-type hepatocytes (Figure 5M–S), whereas the effect of xylitol which bypasses the GK reaction and causes a larger elevation in glycerol 3-P than high glucose (Figure 5J) showed opposite trends by genotype on gene expression. It is noteworthy that glycerol 3-P in incubations with xylitol is measured as a surrogate for changes in phosphate ester intermediates of the pentose cycle including xylulose 5-P which show parallel changes to glycerol 3-P [45].

Cumulatively, hepatocytes from P446L mice on standard rodent diet had similar rates of glucose phosphorylation and glycolysis at basal glucose (5 mM) despite the higher GK-to-GKRP protein ratios and compromised binding affinity of GKRP for GK. But they had lower metabolic rates at elevated glucose and blunted metabolite accumulation and ChREBP-target induction. The similar rates at 5 mM glucose and blunted response at elevated glucose is best explained by compensatory adaptation of total GK activity and gene changes geared to basal glucose homeostasis.

### 3.5 Phenotype of the P446L mouse on high-energy diets

**Figure 6:**
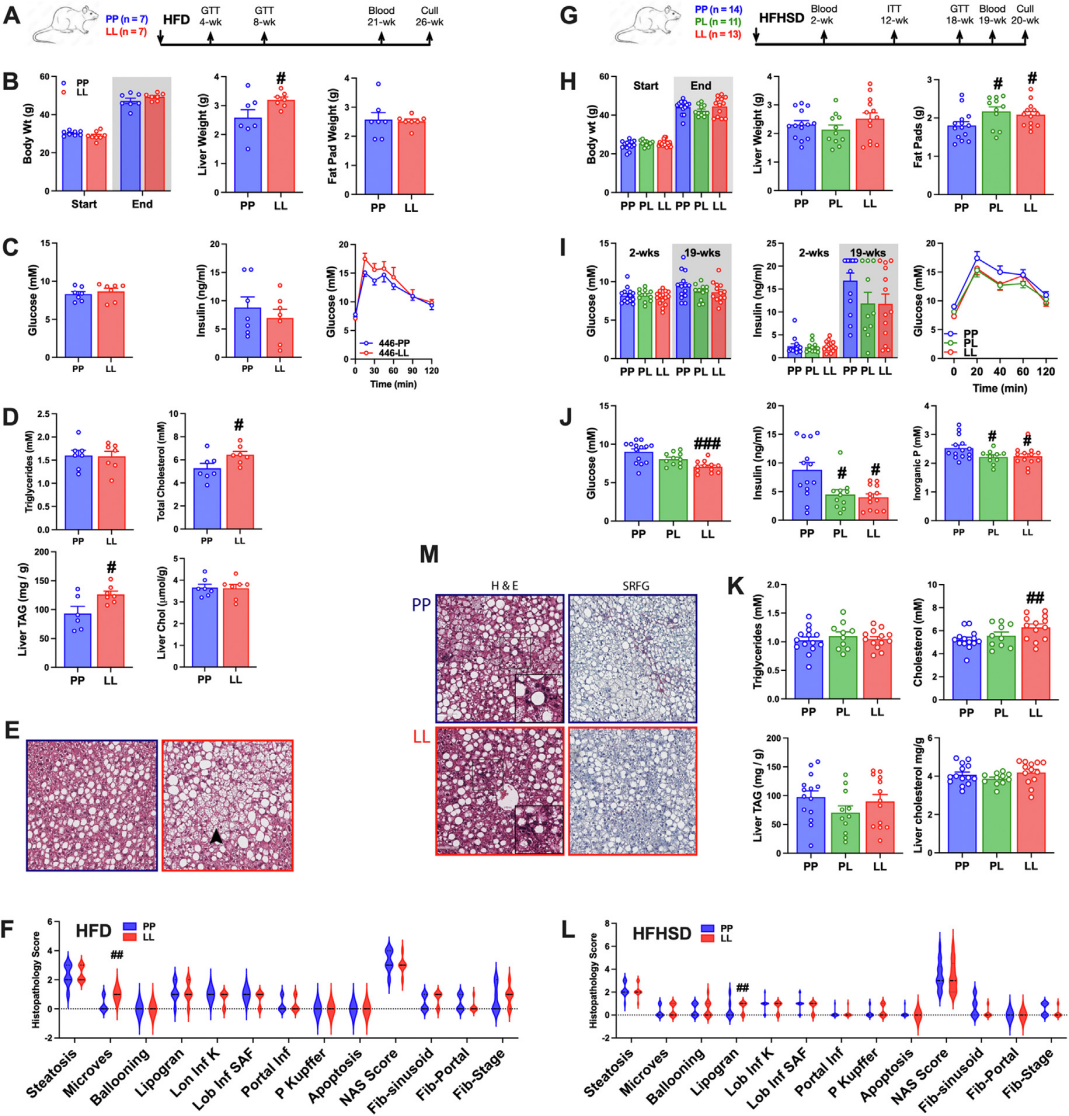
**Higher blood cholesterol and lower fasting blood glucose and insulin in P446L mice on high energy diets**. A-F) Chronic study of P446L mice on HFD: A. Experimental design. B) Body weight, liver weight and epididymis fat pad weight, (n = 7,7). C) No difference in free-feeding blood glucose and insulin (21 wk, n = 7,7) or glucose tolerance (4-wk, GTT, n = 9,9). D) Higher blood cholesterol and liver triglyceride by LL genotype but no difference in blood triglyceride or liver cholesterol (n = 6–7). E) Representative hematoxylin-eosin images showing microvesicular steatosis in LL-genotype (red border). F) Histopathology scores for steatosis, microvesicular steatosis, lipogranulomas, lobular Inflammation, portal Inflammation and fibrosis showing higher microvesicular steatosis by LL genotype (n = 7,7). G-L) Chronic study of P446L mice on HFHSD: G. Experimental design. H. Body weight, liver weight and higher epididymal fat pad wt by LL genotype, n = 11–13. I) Free feeding blood glucose and insulin and glucose tolerance test (GTT), n = 11–14. J) Lower post-prandial blood glucose (2 h food withdrawal) and insulin and inorganic phosphate (Pi) at cull, n = 10–14. K) Higher blood cholesterol in LL genotype but no difference in blood triglycerides or liver triglycerides and cholesterol, n = 10–14. L) Histopathology scores showing higher lipogranuloma and lower fibrosis scores by LL genotype, n = 14,13. M) Representative hematoxylin-eosin images showing lipogranulomas and Sirius red fast green (SRFG) for fibrosis staining. Categorical data statistical differences was by Chi Square test; other analysis was by t-test *#P < 0.05; ##P < 0.01; ###P < 0.001* for genotype effect.

To assess adaptations of the P446L mouse on high-energy diets, mice were fed either a high-fat diet (HFD) with 45% fat by energy or a high-fat high-sugar diet (HFHSD) with the same fat-diet and with sugar (glucose 10% + fructose 5%) in the drinking water in two consecutive studies (Figure 6). Consumption of diet pellets was lower in the HFHSD than HFD study (2.3 ± 0.04 vs 3.0 ± 0.03 g/day).

In the HFD study the LL mice had similar body weight gain (Figure 6A,B) and no significant difference in blood glucose, insulin or glucose tolerance (Figure 6C). There was no difference in blood triglyceride but blood cholesterol was higher (21%) and liver weight and liver triglyceride but not liver cholesterol were also higher in LL mice (Figure 6B,D). Hepatocyte steatosis was evident in all mice, but microvesicular steatosis was more prominent in LL mice (P < 0.002). There was no evidence for hepatocellular ballooning and no difference between genotypes in lobular inflammation, lipogranulomas, Kleiner activity score, sinusoidal fibrosis, portal fibrosis or Kleiner fibrosis stage (Figure 6E,F).

In the HFHSD study (Figure 6G) there was no difference in body weight gain or liver weight but adipose epididymis pad weight was higher in LL mice (Figure 6H). There was no difference by genotype in free-feeding blood glucose and insulin, glucose tolerance (Figure 6I) or insulin sensitivity (not shown). Blood glucose and insulin after 2 h food withdrawal were lower (Figure 6J) and blood Pi (inorganic phosphate) was also lower in LL mice (Figure 6J). Blood triglycerides were not different whereas total blood cholesterol (Figure 6K), LDL-cholesterol (1.67 ± 0.09 vs 2.20 ± 0.14, P < 0.01) and HDL-cholesterol (3.21 ± 0.12 vs 3.74 ± 0.14, P < 0.01) were higher (21%, 32% and 17%, respectively) in LL mice, but there was no difference in liver triglyceride or cholesterol (Figure 6K). Hepatocyte steatosis was evident in most mice, and microvesicular steatosis was not different by genotype (Figure 6L). The LL mice had a higher score (P < 0.006) for lipogranulomas composed of a fat vacuole surrounded by mononuclear cells and macrophages (Figure 6M) but had trends of lower sinusoidal fibrosis (P < 0.058) and Kleiner fibrosis stage (P < 0.051; Figure 6L,M).

Cumulatively, LL mice on HFD had higher blood cholesterol, liver triglycerides and microvesicular steatosis, whereas on HFHSD they had higher adipose pad mass but lower post-prandial glucose and insulin, raised blood cholesterol and raised liver lipogranulomas.

### 3.6 P446L mouse liver transcriptome: altered cholesterol homeostasis on HFHSD

**Figure 7:**
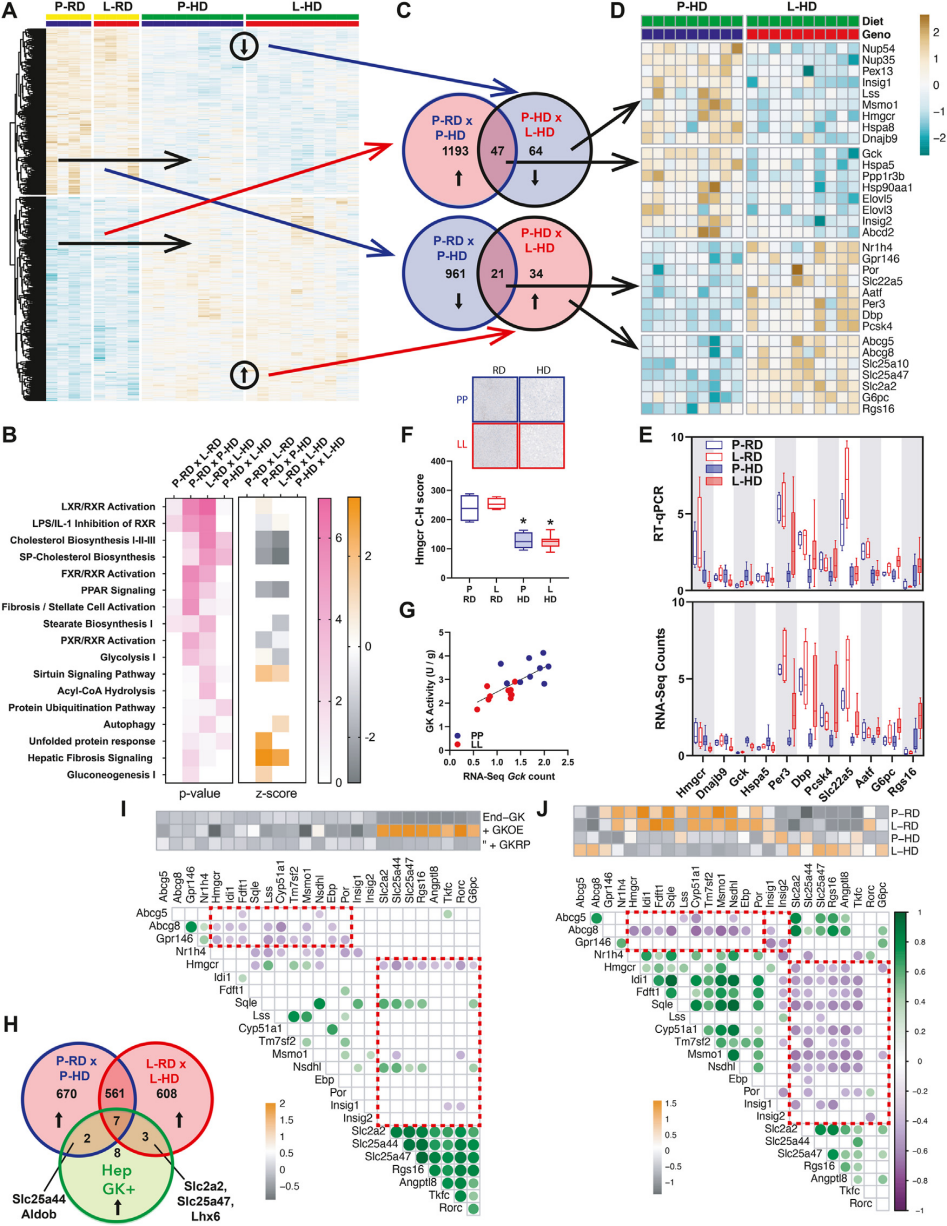
**Liver transcriptome analysis in GKRP**^**P446>L**^**mice on the HFHSD (HD).** A) Heat map for unsupervised clustering of P446L livers of PP and LL genotypes on regular diet (P-RD, P-HD, n = 4,4) or HFHSD (P-HD, L-HD, n = 9,10). B) Ingenuity pathway analysis showing selection of top significantly enriched canonical pathways ranked by P-adjust (FDR). C) Venn diagram for converse gene regulation by diet (P-RD x L-HD) and genotype (P-HD x L-HD). Red up-regulation, blue down-regulation. D) Heat map for selected DEGs from groups in C. E) RT-qPCR validation of DEGs (n = 4,4,10,12) and comparison with RNA-seq counts (n = 4,4,9,10) normalized to P-HD. F) Hmgcr Immunostaining in P446L livers (RD and HD) and cytoplasmic H-scores. n = 4,4 (RD); 9,9 (HD). G. Correlation of GK activity and RNA-seq Gck gene counts (n = 9,10)/. H) Venn diagram for common genes between the hepatocyte transcriptome (up-regulated by GK and down-regulated by GKRP, Figure 2G) and genes up-regulated by diet (P-RD x P-HD; L-RD x L-HD) I) Correlation coefficient matrix for hepatocyte transcriptome (Figure 2: n = 3 hepatocyte preparations, 10 conditions) comparing expression of 17-cholesterol linked identified by the IPA analysis on P446L livers and 8 of 20 genes that were up-regulated by GK-overexpression and counter-regulated by GKRP in the hepatocyte study (Figure 2) showing significant (P < 0.05) positive correlations (green) and negative correlations (purple). The Z-score shows up-regulation orange, down regulation grey. J) Correlation coefficient matrix for P446L mouse liver transcriptomes (P-RD, P-RD, P-HD, L-HD, n = 27) for the same genes as in (H). The Z-score shows relative expression by experimental group (P-RD, L-RD, P-HD and L-HDL-HD).

Analysis of the P446L liver transcriptome in PP, PL and LL genotypes on HFHSD by unsupervised clustering identified 2 groups with either PP or LL genotypes and with PL distributed in the 2 groups. Subsequent analysis excluded the PL genotype and compared PP and LL genotypes on the HFHSD (P-HD, L-HD) and on the regular diet (P-RD, L-RD) (Figure 7A). On the HFHSD there were 111 down-regulated and 55 up-regulated genes by LL genotype (L-HD x P-HD) of which only one gene (*Nup54*, a nucleoporin) was in common with the DEGs on RD.

Pathway enrichment analysis by EnrichGO or Ingenuity Pathway Analysis (IPA) identified cholesterol biosynthesis and LXR activation as the top regulated pathways by LL genotype (L-HD x P-HD). Key DEGs included repression of *Hmgcr, Lss, Msmo1, Insig1, Insig2, Fgf1* and induction of *Por*. There were ∼2000 DEGs between the HD and RD transcriptomes for each genotype, and the top enriched pathways by IPA were cholesterol biosynthesis and lipid signalling (LXR, FXR, PPAR, PXR) which had negative and positive Z-scores, respectively (Figure 7B). To determine whether the DEGs by LL genotype (L-HD x P-HD) represent a blunted response to diet, we compared the up-regulated and down-regulated genes by diet (P-HD x P-RD) with the conversely regulated DEGs by LL-genotype (L-HD x P-HD) (Figure 7C). This showed that 47 of 111 down-regulated genes and 21 of 55 up-regulated genes by LL-genotype (HD) represent a blunted response to diet. Selected DEGs from the 4 groups are shown in the heat map (Figure 7D). In addition to cholesterol biosynthesis, other cholesterol homeostasis genes include up-regulation of *Abcg5* and *Abcg8,* involved in biliary sterol secretion [46], *Gpr146,* linked to blood cholesterol homeostasis in man and mouse [47] and *Nr1h4*, the bile acid receptor. Genes from the 4 groups were validated by RT-qPCR (Figure 7E).

### 3.7 GK-upregulated genes correlate negatively with *Hmgcr* in P446L liver and hepatocytes

Comparison of DEGs by LL-genotype (HD) with genes linked to GK excess from the hepatocyte study (Figure 2) identified 5 common genes: *Slc25a47, Slc2a2, G6pc, Rgs16 (upregulated)* and *Hmgcr* (down-regulated). Immunostaining for HMGCR protein showed lower protein in HD than RD groups but no difference by genotype (Figure 7F). The latter may be linked to repression of *Insig1* and *Insig2* which regulate HMGCR protein stability and uncouple changes in *Hmgcr* mRNA and protein [48]. *Gck* transcript was increased by HFHSD and repressed by LL-genotype and correlated with GK activity (Figure 7F,G).

To explore gene changes linked to GK excess we compared DEGs up-regulated by diet (P-RD x P-HD and L-RD x L-HD) with DEGs induced by GK expression in hepatocytes (Figure 2H). There were 7 common genes up-regulated in both genotypes (*Angptl8, Pklr, Tkfc, Rgs16, Slc17a4, Hcn3, Rnf144b*), 2 unique to PP (*Slc25a44, Aldob*) and 3 to LL (*Slc25a47, Slc2a2, Lhx6*) (Figure 7H).

Correlation analysis of genes linked to GK-excess in hepatocytes (from Figure 2) with genes linked to cholesterol biosynthesis [49] or homeostasis from the IPA (Figure 7B) is shown for both the hepatocyte transcriptome (Figure 7I) and P446L mouse liver (Figure 7J). In the hepatocyte study there were significant negative correlations between *Abcg5*, *Abcg8, Gpr146* and cholesterol biosynthesis genes (n = 18) and negative correlations between *Hmgcr* and genes induced by GK excess (n = 8). Likewise, for P446L mouse liver (Figure 7J) there were negative correlations between *Abcg5, Abcg8* and cholesterol biosynthesis genes (n = 12) or *Insig1,2* (n = 3) and negative correlations between cholesterol biosynthesis genes and genes induced by GK excess (n = 51). Cumulatively, the inverse correlations between *Hmgcr* and either cholesterol excretion-linked genes or GK-inducible genes in both the hepatocyte model of varying GK-to-GKRP and P446L mouse liver support a mechanism linking GK-excess to cholesterol homeostasis.

### 3.8 Blood glucose and cholesterol in the P446L mouse correlate inversely with GK-linked genes

**Figure 8:**
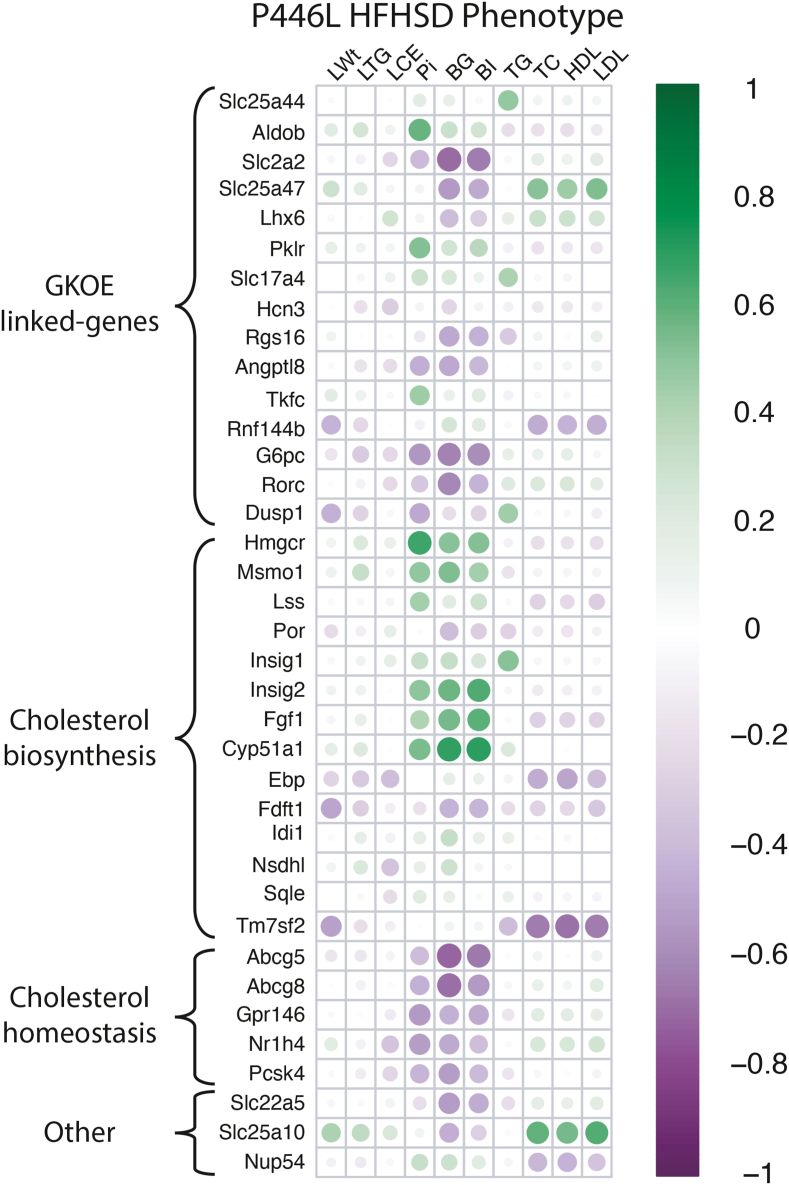
**Correlation of liver transcripts with phenotypic traits in the P446L mouse on the HFHSD.** A) Correlation of phenotypic traits: liver weight, triglyceride, cholesterol (LWt, LTG, LCE) and blood metabolites with corresponding gene counts from the liver transcriptome for the P446L mouse (PP,LL, n = 9/10) on the HSFSD showing: (i) Blood glucose (BG) and blood insulin (BI) correlated negatively with genes induced by GK excess (*Scl2a2, Slc25a47, Rgs16, G6pc, Rorc*) in the hepatocyte transcriptome (Figure 2). (ii) Blood glucose and insulin correlated positively with *Hmgcr* and other genes linked to cholesterol biosynthesis (*Msmo1, Insig2, Cyp51a1*) and negatively with genes linked to cholesterol excretion (*Abcg5, Abcg8*). (iii) Inorganic phosphate (Pi) correlated negatively with *G6*pc; (iv) Blood cholesterol (TC, HDL, LDL) correlated positively with *Slc25a47* and *Slc25a10* and negatively with *Tm7sf2*).

Correlation analysis of phenotype traits (blood glucose, Pi, cholesterol) of the P446L mouse on HFHSD with liver transcriptome counts for genes linked to GK excess or cholesterol homeostasis (Figure 8) shows that blood glucose correlated negatively with genes linked to GK-excess (*Slc2a2, Slc25a47, Angptl8, Rgs16, Rorc, G6pc*) and cholesterol excretion (*Abcg5, Abcg8*) and positively with genes linked to cholesterol biosynthesis (*Hmgcr, Msmo1, Cyp51a1, Insig1*), supporting a link between GK-to-GKRP excess and blood glucose and cholesterol homeostasis. Blood inorganic phosphate (Pi) correlated positively with cholesterol biosynthesis genes (*Hmgcr, Msmo1, Cyp51a1, Insig1*) and negatively with *G6pc*, which generates Pi from glucose 6-P supporting a role for *G6pc* induction in hepatic Pi homeostasis [50,51]. Blood cholesterol (total, HDL, LDL) correlated positively with the *Slc25a47* transporter. Common genetic variation at the human *SLC25A47* locus associates with raised blood total and LDL cholesterol and lower fasting blood glucose in man (CMDKP: hugeamp.org).

## 4 Discussion

### 4.1 Mouse GKRP protein phenocopies the human GKRP P446 > L substitution

The P446 > L substitution has qualitatively similar effects for mouse and human GKRP when GKRP is expressed at physiological levels relative to GK. Two key properties of the 446 L variant are compromised protein expressivity and a lower affinity for sequestering GK in the nucleus. The latter is not contingent on the lower expressivity. However, the lower expressivity is best explained by the lower affinity for GK based on the following considerations. First, overexpression of GK causes increased nuclear sequestration of GKRP. This manifests in both hepatocytes and heterologous cells (FTO2B and HeLa) and for both GKRP:446 P and 446 L and concurs with the small difference in affinity for GK between GKRP-446 P and 446 L. Second, GK overexpression stabilizes GKRP protein as determined by immunoblotting. Thirdly, cytoplasmic GKRP staining is barely detectable in mouse liver in either wild-type or the P446L mouse which has very low levels of GKRP protein in the LL-genotype. This can be explained by the very slow turnover rate of GKRP in mouse liver *in vivo* [52] and a requirement for nuclear sequestration of GKRP for stabilization.

Small differences between mouse and human GKRP in the magnitude of the effect of the P446 > L substitution on glucose-induced translocation of GK concur with the higher affinity of human GKRP compared with rodent GKRP for GK [25] and with the higher nuclear sequestration of both GKRP and GK in hepatocytes transfected with human compared with mouse GKRP. It may also explain the similar sequestration of GK in the nucleus in human (446 PP genotype) compared with mouse liver despite the lower GK and GKRP protein levels. The question whether rodent GKRP adequately models the human-GKRP:P446 > L substitution had remained unsettled based on two considerations. First, significant differences between GKRP:446 P and 446 L in GK kinetic studies were found for human [18] but not rat GKRP [24]. Second, a study expressing chimaeric fluorescent-GKRP constructs (446 P/L) in wild-type mouse hepatocytes found similar nuclear GK sequestration for rat 446 L and 446 P despite lower sequestration for human 446 L [19]. Two points are noteworthy: first, differences in nuclear GK sequestration between 446 L and 446 P are small and critically dependent on the GK-to-GKRP ratio; second, because of the higher affinity for GK of human GKRP, differences between 446 L and 446 P manifest at different GKRP-to-GK ratios necessitating transfections over a range of GK-to-GKRP ratios. We infer that mouse GKRP:P446 > L substitution phenocopies the human GKRP:P446 > L despite the higher affinity of human GKRP for GK. For both species there is lower expressivity of GKRP:446 L and lower nuclear GK sequestration.

### 4.2 Lower liver GK and GKRP protein in human rs1260326-446LL genotype and in the P446L mouse

Human NAFLD tissue homozygous for the 446 L allele (rs1260326,TT) had lower GKRP and GK protein levels and lower ratios of nuclear GK/nuclear GKRP, which concurs with the lower expressivity of the GKRP-446 L variant and the lower nuclear GK sequestration. It also concurs with the P446L mouse which has 70–90% reduction in GKRP protein and 40–50% reduction in GK protein. The very low GKRP protein levels in young mice on regular diet was surprising given the modestly lower expressivity of the 446 L variant when transfected in hepatocytes at high mRNA transcript levels to attain physiological GKRP protein levels within ∼24 h culture. It is best explained by the very slow turnover rate of GKRP protein in mouse liver *in vivo* (t_1/2_ 6.5 days) compared with either GK (t_1/2_ 4.6 days) or the average value (t_1/2_ < 1 d) for mouse liver protein [52]. The P446L mouse had a reduction in total GK protein and activity but a relative excess of GK-to-GKRP protein ∼2-4-fold compared with wild-type mice and shares some similarities with *Gckr*-knock-out mice [32,33] which have ∼50% reduction in GK but maintain glucose homeostasis on a standard diet. It is widely assumed that the low GK protein in *Gckr*-deficient states is consequent to a stabilizing role of GKRP. However direct evidence for such a mechanism is lacking. In the present study in hepatocytes overexpressing GK and GKRP there was evidence for GK stabilizing GKRP but weaker non-significant trends for GKRP stabilizing GK. The lower GK protein levels in the P446L mouse may be in part due to transcriptional regulation analogous to induction of *G6pc* [51,52]. The metabolic studies on hepatocytes from the P446L mouse showed that glucose disposal rates were identical at basal glucose (5 mM) but lower for the 446LL genotype at elevated glucose. Likewise, the induction of ChREBP-β and its target genes during high-glucose challenge was also blunted. The lower metabolic capacity of 446LL hepatocytes at high glucose challenge indicates that despite the relative GK-to-GKRP excess there is effective adaptation to maintain basal glucose homeostasis on the regular diet. The mice on the HFHSD (both wild-type and 446LL) had a ∼2-fold increase in GK and an increase in GKRP. An increase in GK activity on a high-fat diet has been reported and concurs with the raised insulin [53]. Given the established role of insulin in promoting hepatic GK expression [11] the concurrent increase in GKRP is at least in part consequent to the stabilizing effect of GK.

### 4.3 Lower blood glucose and insulin in the P446L mouse on the HSHSD

The *GCKR* locus is one of ∼240 loci that associate with blood glucose or type 2 diabetes risk [1,54]. The majority of the associated variants are intronic and of the ∼20 associated missense variants, few have been validated in mouse models [55,56]. The human *GCKR*-446 L allele is associated with higher blood triglycerides and lower blood glucose, with a smaller effect size on blood glucose (<4%) than on triglycerides (8–25%) [[3], [4], [5]] and with the widely held assumption that it represents increased hepatic conversion of glucose to triglyceride [5]. The P446L mouse on the HFHSD had lower post-prandial blood glucose and insulin without an increase in blood triglyceride. The negative correlation between blood glucose and transcript levels of genes that are induced by GK overexpression supports a role for functional GK excess in the lower blood glucose. Two mechanisms can be considered consequent to the higher GK-to-GKRP protein ratio increased hepatic glucose uptake and lower hepatic gluconeogenesis. The latter is supported by the higher rates of endogenous glucose production in people with *GCK* inactivating mutations [57]. Lower endogenous glucose production in hyperglycaemic clamps was also found in association with *GCKR* rs780094^C > T^ [5]. The negative correlation with blood glucose and inorganic phosphate (Pi) of the *G6pc* transcript which encodes the final enzyme in hepatic glucose production, is best explained by the role of this enzyme in hepatic phosphate homeostasis [51,52]. The lower blood insulin may be consequent to the lower blood glucose or the lower hepatic GK protein because lower insulin was found in liver-selective GK-deficient models [58]. Cumulatively, the P446L mouse on the HFHSD replicates the lower blood glucose and insulin but not the raised blood triglyceride found in genome-wide associations with the *GCKR* locus.

### 4.4 Raised blood cholesterol and changes in cholesterol-linked genes in the P446L mouse

The P446L mouse on the high-energy diets had raised blood cholesterol without raised blood triglycerides and with a liver transcriptome characterized by altered cholesterol homeostasis. This included repression of cholesterol biosynthesis enzymes (*Hmgcr, Msmo1, Lss*) and the cholesterol regulators *Insig1* and *Insig2* which function as sterol sensors and have a dual role in anchoring the transcription factor SREBP-SCAP to the ER membrane and promoting ubiquitination and degradation of the Hmgcr protein, the limiting enzyme of cholesterol biosynthesis. It also included induction of *Abcg5 and Abcg8* encoding cotransporters of biliary sterol excretion [46] and *Gpr146* which is linked to blood cholesterol in man and mouse [47]. *Hmgcr* gene repression was identified by RNA-sequencing in hepatocytes overexpressing GK, indicating a candidate link from the GK-to-GKRP ratio to altered cholesterol regulation. Moreover, in both the hepatocytes expressing varying GK-to-GKRP ratios and in the P446L mouse, *Hmgcr* transcript levels correlated positively with *Msmo1* and *Lss* and negatively with several genes induced by GK excess including putative ChREBP target genes (*G6pc, Rgs16, Angptl8, Slc2a2*) and various mitochondrial transporters including *Slc25a47* which was reported to correlate negatively with cholesterol biosynthesis [42]. The lack of difference in Hmgcr protein in the P446L mouse despite lower transcript levels may be linked to repression of *Insig1* and *Insig2* because down-regulation of these genes leads to increased Hmgcr protein compared with mRNA levels [48]. Common human variants for *HMGCR, INSIG1, INSIG2, ABCG5, ABCG8* and *GPR146* are associated with blood cholesterol levels (https://hugeamp.org), although with modest effects for *HMGCR* variants [59]. This concurs with the complexity of *HMGCR* regulation whereby sterols inhibit transcription whereas nonsterol isoprenoids inhibit translation and both metabolites promote proteasomal degradation [60].

The mechanisms linking molar GK-to-GKRP excess with altered cholesterol homeostasis remain to be elucidated. An increase in both blood cholesterol and triglyceride occurs in *G6pc* deficiency in man and mouse [[61], [62], [63]]. This is a more severe phenotype of hepatic glucose 6-P accumulation and ChREBP activation because of the major role of glucose 6-phosphatase in hepatic phosphate homeostasis [50], [51], [52] as shown by the greater accumulation of glucose 6-P by inhibition of glucose 6-P hydrolysis relative to GK activation with pharmacological activators (Figure 5). In the hepatocyte model of GK-to-GKRP excess challenged with high glucose, the repression of *Hmgcr* correlated negatively with multiple genes induced by metabolite accumulation including the hepatokine *Angptl8* and the transcriptional regulator *Rorc*, linked to cholesterol metabolism [64]. Because significant repression of *Hmgcr* in the P446L mouse occurred after the HFHSD and together with induction of genes characterized by GK excess (*G6pc, Rgs16, slc2a2, Slc25a47*), altered cholesterol homeostasis occurs in conjunction with ChREBP activation. Elevated expression of cholesterol biosynthesis genes by ChREBP knock-down in a metabolically challenged state has been reported [65].

### 4.5 Blood triglycerides in the P446L mouse compared with human *GCKR* associations

The lack of raised blood triglycerides despite raised blood cholesterol and lower blood glucose in the P446L mouse was confounding, given that the human *GCKR* variant associates with a greater effect on blood triglycerides than on blood cholesterol [7,8] or blood glucose [[3], [4], [5]]. Mouse models of *Gckr*-deficiency which are essentially models of GK-to-GKRP excess likewise did not have raised blood triglycerides [33]. Amongst *Gck*-transgenic models, which represent GK-excess at endogenous GKRP, raised blood and liver triglycerides was found in 12-month old mice which had raised blood glucose and insulin [66] but not in young insulin-deficient mice with a greater GK-excess [67]. Additionally, there was no increase in liver triglyceride in a *Gck*-transgenic model on a HFD, which had lower blood insulin in conjunction with 2-fold GK excess [53]. Cumulatively, in mouse genetic models of relative GK-to-GKRP excess, the raised blood and liver triglycerides in conjunction with raised blood insulin and glucose [66] implicates insulin resistance. In human *GCKR* association studies it was noted at the outset that the *GCKR* effect on blood triglycerides was greater in populations with higher body mass index [4]. This was later corroborated by a larger effect of the *GCKR* variant on blood [68] and liver [69] triglycerides in obesity and type 2 diabetes [70,71]. An alternative hypothesis to gene–phenotype interactions is “quantile-dependent expressivity” where the genetic difference on triglycerides depends on the magnitude of the triglyceride concentration in the population [72]. In the context of relative associations of the *GCKR* variant with blood triglycerides and cholesterol, studies on children corroborate the above hypotheses [[73], [74], [75]] and in healthy paediatric cohorts with substantially lower triglyceride concentration than in the adult population the *GCKR* variant associates with raised blood cholesterol but not triglyceride [73]. Raised blood triglycerides occurs through raised hepatic secretion and/or impaired extrahepatic clearance, and clinically high blood triglycerides generally reflect compromised triglyceride clearance as occurs in obesity [76]. The lack of increased blood triglycerides in the P446L mouse could be linked to efficient extrahepatic triglyceride clearance, because of deficiency in cholesteryl ester transfer protein [77] or other mechanisms. The raised liver triglyceride and microvesicular steatosis on the HFD and the increased fat pad mass on the HFHSD could be consistent with this interpretation. Raised blood triglycerides occurs in human *G6PC* deficiency and in mouse models of *G6pc* deficiency [[61], [62], [63]]. This represents an analogous but more severe hepatic dysregulation of glucose 6-P than relative GK-to-GKRP excess. The hypertriglyceridemia in mouse models of *G6pc* deficiency is associated with impaired extrahepatic clearance [62].

The liver produces the hepatokine ANGPTL8 which functions as an inhibitor of triglyceride clearance by oxidative tissues thereby promoting raised blood triglycerides and/or diversion of triglyceride to adipose sites [78,79]. In man circulating ANGPTL8 levels correlate with obesity and interestingly also with the human *GCKR* rs1260326 variant [80]. The current finding that hepatocyte expression of *Angptl8* correlates with GK excess over GKRP provides an explanation for a causal mechanism between the *GCKR* variant and the raised blood ANGPTL8 levels in man [80]. ANGPTL8 thereby represents one candidate, consequent to GK excess that can amplify blood triglyceride levels in association with the *GCKR* variant. However, the possibility that other gene variants at the *GCKR* locus on neighbouring genes that are in linkage disequilibrium can have additional synergistic effect on blood triglycerides needs to be considered.

## Author contributions

L.A. directed the project, conceived and designed the experiments, contributed to the analysis and co-wrote the paper. B.E.F. and S·S.C. conducted the experiments, analyzed the data and co-wrote the paper., D.T. conducted the histopathology analysis and interpretation; K.R., T.M., Z.A.O., H.L.R., Q.M.A., J.M.S., R.J.F., D.M.S., contributed to data collection or analysis. All authors approved the final version prior to submission for publication.

## Data Availability

Figure data is available at data.ncl. RNA-seq data is available at NCBI GEO: GSE228696, GSE228697, GSE228698.

## References

[1] Chen J., Spracklen C.N., Marenne G., Varshney A., Corbin L.J., Luan J. (2021). The trans-ancestral genomic architecture of glycemic traits. Nat Genet.

[2] Dron J.S., Hegele R.A. (2020). Genetics of hypertriglyceridemia. Front Endocrinol.

[3] Sparsø T., Andersen G., Nielsen T., Burgdorf K.S., Gjesing A.P., Nielsen A.L. (2008). The GCKR rs780094 polymorphism is associated with elevated fasting serum triacylglycerol, reduced fasting and OGTT-related insulinaemia, and reduced risk of type 2 diabetes. Diabetologia.

[4] Vaxillaire M., Cavalcanti-Proença C., Dechaume A., Tichet J., Marre M., Balkau B., DESIR Study Group (2008). The common P446L polymorphism in GCKR inversely modulates fasting glucose and triglyceride levels and reduces type 2 diabetes risk in the DESIR prospective general French population. Diabetes.

[5] Orho-Melander M., Melander O., Guiducci C., Perez-Martinez P., Corella D., Roos C. (2008). Common missense variant in the glucokinase regulatory protein gene is associated with increased plasma triglyceride and C-reactive protein but lower fasting glucose concentrations. Diabetes.

[6] Ingelsson E., Langenberg C., Hivert M.F., Prokopenko I., Lyssenko V., Dupuis J. (2010). Detailed physiologic characterization reveals diverse mechanisms for novel genetic Loci regulating glucose and insulin metabolism in humans. Diabetes.

[7] Teslovich T.M., Musunuru K., Smith A.V., Edmondson A.C., Stylianou I.M., Koseki M. (2010). Biological, clinical and population relevance of 95 loci for blood lipids. Nature.

[8] Willer C.J., Schmidt E.M., Sengupta S., Peloso G.M., Gustafsson S., Kanoni S. (2013). Discovery and refinement of loci associated with lipid levels. Nat Genet.

[9] Anstee Q.M., Darlay R., Cockell S., Meroni M., Govaere O., Tiniakos D., EPoS Consortium Investigators (2020). Genome-wide association study of non-alcoholic fatty liver and steatohepatitis in a histologically characterised cohort. J Hepatol.

[10] Van Schaftingen E., Detheux M., Veiga da Cunha M. (1994). Short-term control of glucokinase activity: role of a regulatory protein. Faseb J.

[11] Agius L. (2016). Hormonal and metabolite regulation of hepatic glucokinase. Annu Rev Nutr.

[12] Lloyd D.J., St Jean D.J., Kurzeja R.J., Wahl R.C., Michelsen K., Cupples R. (2013). Antidiabetic effects of glucokinase regulatory protein small-molecule disruptors. Nature.

[13] Veiga-da-Cunha M., Van Schaftingen E. (2002). Identification of fructose 6-phosphate and fructose 1-phosphate binding residues in the regulatory protein of glucokinase. J Biol Chem.

[14] Veiga-da-Cunha M., Sokolova T., Opperdoes F., Van Schaftingen E. (2009). Evolution of vertebrate glucokinase regulatory protein from a bacterial N-acetylmuramate 6-phosphate etherase. Biochem J.

[15] Vandercammen A., Van Schaftingen E. (1993). Species and tissue distribution of the regulatory protein of glucokinase. Biochem J.

[16] Agius L., Peak M., Newgard C.B., Gomez-Foix A.M., Guinovart J.J. (1996). Evidence for a role of glucose-induced translocation of glucokinase in the control of hepatic glycogen synthesis. J Biol Chem.

[17] de la Iglesia N., Mukhtar M., Seoane J., Guinovart J.J., Agius L. (2000). The role of the regulatory protein of glucokinase in the glucose sensory mechanism of the hepatocyte. J Biol Chem.

[18] Beer N.L., Tribble N.D., McCulloch L.J., Roos C., Johnson P.R., Orho-Melander M. (2009). The P446L variant in GCKR associated with fasting plasma glucose and triglyceride levels exerts its effect through increased glucokinase activity in liver. Hum Mol Genet.

[19] Rees M.G., Wincovitch S., Schultz J., Waterstradt R., Beer N.L., Baltrusch S. (2012). Cellular characterisation of the GCKR P446L variant associated with type 2 diabetes risk. Diabetologia.

[20] Zelent B., Raimondo A., Barrett A., Buettger C.W., Chen P., Gloyn A.L. (2014). Analysis of the co-operative interaction between the allosterically regulated proteins GK and GKRP using tryptophan fluorescence. Biochem J.

[21] Brouwers M.C.G.J., Jacobs C., Bast A., Stehouwer C.D.A., Schaper N.C. (2015). Modulation of glucokinase regulatory protein: a double-edged sword?. Trends Mol Med.

[22] López Rodríguez M., Kaminska D., Lappalainen K., Pihlajamäki J., Kaikkonen M.U., Laakso M. (2017). Identification and characterization of a FOXA2-regulated transcriptional enhancer at a type 2 diabetes intronic locus that controls GCKR expression in liver cells. Genome Med.

[23] Morris A.P. (2014). Fine mapping of type 2 diabetes susceptibility loci. Curr Diabetes Rep.

[24] Veiga-da-Cunha M., Delplanque J., Gillain A., Bonthron D.T., Boutin P., Van Schaftingen E. (2003). Mutations in the glucokinase regulatory protein gene in 2p23 in obese French caucasians. Diabetologia.

[25] Brocklehurst K.J., Davies R.A., Agius L. (2004). Differences in regulatory properties between human and rat glucokinase regulatory protein. Biochem J.

[26] Codner G.F., Mianné J., Caulder A., Loeffler J., Fell R., King R. (2018). Application of long single-stranded DNA donors in genome editing: generation and validation of mouse mutants. BMC Biol.

[27] Hardy T., Wonders K., Younes R., Aithal G.P., Aller R., Allison M. (2020). The European NAFLD Registry: a real-world longitudinal cohort study of nonalcoholic fatty liver disease. Contemp Clin Trials.

[28] Kleiner D.E., Brunt E.M., Van Natta M., Behling C., Contos M.J., Cummings O.W., Nonalcoholic Steatohepatitis Clinical Research Network (2005). Design and validation of a histological scoring system for nonalcoholic fatty liver disease. Hepatology.

[29] Alshawi A., Agius L. (2019). Low metformin causes a more oxidized mitochondrial NADH/NAD redox state in hepatocytes and inhibits gluconeogenesis by a redox-independent mechanism. J Biol Chem.

[30] Becker T.C., Noel R.J., Johnson J.H., Lynch R.M., Hirose H., Tokuyama Y. (1996). Differential effects of overexpressed glucokinase and hexokinase I in isolated islets. Evidence for functional segregation of the high and low Km enzymes. J Biol Chem.

[31] Davidson A.L., Arion W.J. (1987). Factors underlying significant underestimations of glucokinase activity in crude liver extracts: physiological implications of higher cellular activity. Arch Biochem Biophys.

[32] Goodman R.P., Markhard A.L., Shah H., Sharma R., Skinner O.S., Clish C.B. (2020). Hepatic NADH reductive stress underlies common variation in metabolic traits. Nature.

[33] Farrelly D., Brown K.S., Tieman A., Ren J., Lira S.A., Hagan D. (1999). Mice mutant for glucokinase regulatory protein exhibit decreased liver glucokinase: a sequestration mechanism in metabolic regulation. Proc Natl Acad Sci USA.

[34] Grimsby J., Coffey J.W., Dvorozniak M.T., Magram J., Li G., Matschinsky F.M. (2000). Characterization of glucokinase regulatory protein-deficient mice. J Biol Chem.

[35] Park J.M., Kim T.H., Jo S.H., Kim M.Y., Ahn Y.H. (2015). Acetylation of glucokinase regulatory protein decreases glucose metabolism by suppressing glucokinase activity. Sci Rep.

[36] Watanabe H., Inaba Y., Kimura K., Matsumoto M., Kaneko S., Kasuga M. (2018). Sirt2 facilitates hepatic glucose uptake by deacetylating glucokinase regulatory protein. Nat Commun.

[37] Mukhtar M., Stubbs M., Agius L. (1999). Evidence for glucose and sorbitol-induced nuclear export of glucokinase regulatory protein in hepatocytes. FEBS Lett.

[38] Ma L., Robinson L.N., Towle H.C. (2006). ChREBP∗Mlx is the principal mediator of glucose-induced gene expression in the liver. J Biol Chem.

[39] Jeong Y.S., Kim D., Lee Y.S., Kim H.J., Han J.Y., Im S.S. (2011). Integrated expression profiling and genome-wide analysis of ChREBP targets reveals the dual role for ChREBP in glucose-regulated gene expression. PLoS One.

[40] Poungvarin N., Chang B., Imamura M., Chen J., Moolsuwan K., Sae-Lee C. (2015). Genome-wide analysis of ChREBP binding sites on male mouse liver and white adipose chromatin. Endocrinology.

[41] Darbani B. (2021). Genome evolutionary dynamics meets functional Genomics: a case story on the identification of SLC25A44. Int J Mol Sci.

[42] Bresciani N., Demagny H., Lemos V., Pontanari F., Li X., Sun Y. (2022). The Slc25a47 locus is a novel determinant of hepatic mitochondrial function implicated in liver fibrosis. J Hepatol.

[43] Penn D.J., Zala S.M., Luzynski K.C. (2022). Regulation of sexually dimorphic expression of major urinary proteins. Front Physiol.

[44] Ford B.E., Chachra S.S., Alshawi A., Brennan A., Harnor S., Cano C. (2020). Chronic glucokinase activator treatment activatesliver Carbohydrate response element binding protein and improves hepatocyte ATP homeostasis during substrate challenge. Diabetes Obes Metabol.

[45] Vincent M.F., Van den Berghe G., Hers H.G. (1989). D-xylulose-induced depletion of ATP and Pi in isolated rat hepatocytes. Faseb J.

[46] Sun Y., Wang J., Long T., Qi X., Donnelly L., Elghobashi-Meinhardt N. (2021). Molecular basis of cholesterol efflux via ABCG subfamily transporters. Proc Natl Acad Sci U S A.

[47] Wilkins B.P., Finch A.M., Wang Y., Smith N.J. (2022). Orphan GPR146: an alternative therapeutic pathway to achieve cholesterol homeostasis?. Trends Endocrinol Metabol.

[48] Engelking L.J., Liang G., Hammer R.E., Takaishi K., Kuriyama H., Evers B.M. (2005). Schoenheimer effect explained—feedback regulation of cholesterol synthesis in mice mediated by Insig proteins. J Clin Invest.

[49] Sharpe L.J., Brown A.J. (2013). Controlling cholesterol synthesis beyond 3-hydroxy-3-methylglutaryl-CoA reductase (HMGCR). J Biol Chem.

[50] Aiston S., Green A., Mukhtar M., Agius L. (2004). Glucose 6-phosphate causes translocation of phosphorylase in hepatocytes and inactivates the enzyme synergistically with glucose. Biochem J.

[51] Agius L. (2016). Dietary carbohydrate and control of hepatic gene expression: mechanistic links from ATP and phosphate ester homeostasis to the carbohydrate- response element-binding protein. Proc Nutr Soc.

[52] Arden C., Petrie J.L., Tudhope S.J., Al-Oanzi Z., Claydon A.J., Beynon R.J. (2011). Elevated glucose represses liver glucokinase and induces its regulatory protein to safeguard hepatic phosphate homeostasis. Diabetes.

[53] Shiota M., Postic C., Fujimoto Y., Jetton T.L., Dixon K., Pan D. (2001). Glucokinase gene locus transgenic mice are resistant to the development of obesity-induced type 2 diabetes. Diabetes.

[54] Mahajan A., Taliun D., Thurner M., Robertson N.R., Torres J.M., Rayner N.W. (2018). Fine-mapping type 2 diabetes loci to single-variant resolution using high-density imputation and islet-specific epigenome maps. Nat Genet.

[55] Fan Y., Wolford B.N., Lu H., Liang W., Sun J., Zhou W. (2021). Type 2 diabetes sex-specific effects associated with E167K coding variant in TM6SF2. iScience.

[56] Sachse G., Haythorne E., Hill T., Proks P., Joynson R., Terrón-Expósito R. (2021). The KCNJ11-E23K gene variant hastens diabetes progression by impairing glucose-induced insulin secretion. Diabetes.

[57] Tappy L., Dussoix P., Iynedjian P., Henry S., Schneiter P., Zahnd G. (1997). Abnormal regulation of hepatic glucose output in maturity-onset diabetes of the young caused by a specific mutation of the glucokinase gene. Diabetes.

[58] Magnuson M.A., She P., Shiota M. (2003). Gene-altered mice and metabolic flux control. J Biol Chem.

[59] Plenge R.M., Scolnick E.M., Altshuler D. (2013). Validating therapeutic targets through human genetics. Nat Rev Drug Discov.

[60] Schumacher M.M., DeBose-Boyd R.A. (2021). Posttranslational regulation of HMG CoA reductase, the rate-limiting enzyme in synthesis of cholesterol. Annu Rev Biochem.

[61] Peng W.T., Pan C.J., Lee E.J., Westphal H., Chou J.Y. (2009). Generation of mice with a conditional allele for G6pc. Genesis.

[62] Hoogerland J.A., Peeks F., Hijmans B.S., Wolters J.C., Kooijman S., Bos T. (2021). Impaired very-low-density lipoprotein catabolism links hypoglycemia to hypertriglyceridemia in glycogen storage disease type ia. J Inherit Metab Dis.

[63] La Rose A.M., Groenen A.G., Halmos B., Bazioti V., Rutten M.G.S., Krishnamurthy K.A. (2022). Increased atherosclerosis in a mouse model of glycogen storage disease type 1a. Mol Genet Metab Rep.

[64] Zou H., Yang N., Zhang X., Chen H.W. (2022). RORγ is a context-specific master regulator of cholesterol biosynthesis and an emerging therapeutic target in cancer and autoimmune diseases. Biochem Pharmacol.

[65] Zhang D., Tong X., VanDommelen K., Gupta N., Stamper K., Brady G.F. (2017). Lipogenic transcription factor ChREBP mediates fructose-induced metabolic adaptations to prevent hepatotoxicity. J Clin Invest.

[66] Ferre T., Riu E., Franckhauser S., Agudo J., Bosch F. (2003). Long-term overexpression of glucokinase in the liver of transgenic mice leads to insulin resistance. Diabetologia.

[67] Ferre T., Pujol A., Riu E., Bosch F., Valera A. (1996). Correction of diabetic alterations by glucokinase. Proc Natl Acad Sci U S A.

[68] Cole C.B., Nikpay M., Lau P., Stewart A.F., Davies R.W., Wells G.A. (2014). Adiposity significantly modifies genetic risk for dyslipidemia. J Lipid Res.

[69] Stender S., Kozlitina J., Nordestgaard B.G., Tybjærg-Hansen A., Hobbs H.H., Cohen J.C. (2017). Adiposity amplifies the genetic risk of fatty liver disease conferred by multiple loci. Nat Genet.

[70] Kimura M., Iguchi T., Iwasawa K., Dunn A., Thompson W.L., Yoneyama Y. (2022). En masse organoid phenotyping informs metabolic-associated genetic susceptibility to NASH. Cell.

[71] Simons N., Dekker J.M., van Greevenbroek M.M., Nijpels G., Hart L.M., van der Kallen C.J. (2016). A common gene variant in glucokinase regulatory protein interacts with glucose metabolism on diabetic dyslipidemia: the combined CODAM and hoorn studies. Diabetes Care.

[72] Williams P.T. (2020). Gene-environment interactions due to quantile-specific heritability of triglyceride and VLDL concentrations. Sci Rep.

[73] Lee H.J., Jang H.B., Kim H.J., Ahn Y., Hong K.W., Cho S.B. (2015). The dietary monounsaturated to saturated fatty acid ratio modulates the genetic effects of GCKR on serum lipid levels in children. Clin Chim Acta.

[74] Hovsepian S., Javanmard S.H., Mansourian M., Tajadini M., Hashemipour M., Kelishadi R. (2018). Relationship of lipid regulatory gene polymorphisms and dyslipidemia in a pediatric population: the CASPIAN III study. Hormones (Basel).

[75] Shen Y., Xi B., Zhao X., Cheng H., Hou D., Wu L. (2013). Common genetic variants associated with lipid profiles in a Chinese pediatric population. Hum Genet.

[76] Lewis G.F., Xiao C., Hegele R.A. (2015). Hypertriglyceridemia in the genomic era: a new paradigm. Endocr Rev.

[77] Barter P.J., Brewer H.B., Chapman M.J., Hennekens C.H., Rader D.J., Tall A.R. (2003). Cholesteryl ester transfer protein: a novel target for raising HDL and inhibiting atherosclerosis. Arterioscler Thromb Vasc Biol.

[78] Wang Y., Quagliarini F., Gusarova V., Gromada J., Valenzuela D.M., Cohen J.C. (2013). Mice lacking ANGPTL8 (Betatrophin) manifest disrupted triglyceride metabolism without impaired glucose homeostasis. Proc Natl Acad Sci U S A.

[79] Oldoni F., Cheng H., Banfi S., Gusarova V., Cohen J.C., Hobbs H.H. (2020). ANGPTL8 has both endocrine and autocrine effects on substrate utilization. JCI Insight.

[80] Oldoni F., Bass K., Kozlitina J., Hudson H., Shihanian L.M., Gusarova V. (2021). Genetic and metabolic determinants of plasma levels of ANGPTL8. J Clin Endocrinol Metab.

