# Supplementary material for: The GCKR-P446L gene variant predisposes to raised blood cholesterol and lower blood glucose in the P446L mouse-a model for GCKR rs1260326
Source: Mol Metab. 2023 Apr 7;72:101722. doi: 10.1016/j.molmet.2023.101722 (PMC10182400; doi:10.1016/j.molmet.2023.101722)
Supplement: Multimedia component 1 [file mmc1.docx]

**Table S1 Primers and Reagents**

| **Primers** | ***Forward*** | ***Reverse*** | Notes |
| --- | --- | --- | --- |
| H GCKR 1 | AACCTCTTTACACCTACCTC | GACAATCACTCTCTTCTTCC | Fig-S1 |
| H GCKR 2 | CAGATGATATTCGGGCTGCT | CAGCAAGGCGATGGGTAT | Fig-S1 |
| Gckr ex14-15 | TGACTTCCTGACTTCTGTTC | TACTTCTGCGAGGTTATCAT | Fig-S1,3 |
| Gckr-t2 | GGAGCTACGTTCAGAAGTTCCA | TGTCCTGAGAACCTTGTATTCAACA | Fig-S2 |
| Gckr-X1 | TACCTCATTGCAGGGGGTGA | CAAAGGGCGCCTTCTTCAG | Fig-S2 |
| Gckr-X2 | CTGGGGAATGGGAGCGACTG | CTCCTTCAGCACTTCCTGGAC | Fig-S2 |
| Gckr ex17-del | CAAGTGGGTGTTGAATACAGTGA | CCAGCATGTGGTTCTGTAGG | T1,X1,X2(Fig-S2) |
| Gckr ex7-del | CGGAATCGAGGAGCTGAA | CCACGGAAATGCCTATAACG | T1,T2,X2(Fig-S2) |
| Gckr ex2-del | CTGGGGCAGTGTGATGCT | CTGGTAGGTGGGCATGATTT | T1,T2,X1(Fig-S2) |
| H SIRT2 | agagccagacccctctcac | catgtctgcttctccaccag | Fig-S3 |
| Gapdh | GACAATGAATACGGCTACAGCA | GGCCTCTCTTGCTCAGTGTC | House keeping |
| Rplpo | AGGCGTCCTCGTTGGAGT | AAAAGTTGGATGATCTTGAGGAAG | House keeping |
| TBP | GGCGGTTTGGCTAGGTTT | TCTGGGTTATCTTCACACACCA | House keeping |
| Gck | ACAGTCTCCTTCATATACCTCCAC | CTCTATCCTCTGGCATCTCCT | Fig-3 |
| Gpd2 | ACTACCTGAGTTCTGACGTTGAAG | TAACAAGGGGACGGATACCA | Fig-5 |
| ChREBP-β | TCTGCAGATCGCGTGGAG | CTTGTCCCGGCATAGCAAC | Fig-5 |
| Pklr | CTAATCTGGCTGTTCTCTTG | \| TGCTTCTGATCTGTATGTGG \| \| --- \| | Fig-5 |
| Txnip | CTTGCGCTATGAAGACACACTT | GGCCTCATGATCACCATCTC | Fig-5 |
| Fgf21 | AGATGGAGCTCTCTATGGATCG | GGGCTTCAGACTGGTACACAT | Fig-5 |
| Fasn | CAACATGGGACACCCTGAG | GTTGTGGAAGTGCAGGTTAGG | Fig-S5 |
| Rgs16 | GGCCAGTAAGCATAACAAAGAGA | TCAGCAGCAAATCGAAAGAC | Fig-S5 |
| Angptl8 | GCCCACCAAGAATTTGAGAC | GCCAGTGAGAGCCCATAAGA | Fig-S5 |
| G6pc | TCTGTCCCGGATCTACCTTG | GAAAGTTTCAGCCACAGCAA | Fig-S5, Fig-5 |
| Aldob | GAAACCGCCTGCAAAGGATAA | GAGGGTCTCGTGGAAAAGGAT | Fig-S5 |
| Slc2a2 | TTACCGACAGCCCATCCT | TGAAAAATGCTGGTTGAATAGTAAAA | Fig-S5 |
| Slc25a47 | TGTGCTGTCCATGGATTTTG | GAGGGTAGCCCACAGCAAC | Fig-S5 |
| Rorc | AGAAGACCCACACCTCACAAA | CCTCACAGGTGATAACCCCG | Fig-S5 |
| Hmgcr | CGTAAGCGCAGTTCCTTCC | TTGTAGCCTCACAGTCCTTGG | Fig-S5, Fig-7 |
| Dnajb9 | CACAAAGATGCCTTTTCTACCG | TTAAACTTTTCAGCTTAATGACGTG | Fig-7 |
| Hspa5 | CTGAGGCGTATTTGGGAAAG | TCATGACATTCAGTCCAGCAA | Fig-7 |
| Per3 | GTGAAGCCAGTGGCAGAGA | TGAGAGGAAGAAAAGTCCTTCTG | Fig-7 |
| Dbp | GCATTCCAGGCCATGAGACT | CCAGTACTTCTCATCCTTCTGT | Fig-7 |
| Pcsk4 | ACGACTGAAGAAAGATCCCAAG | CACCAGGGAGCGTTTCAC | Fig-7 |
| Slc22a5 | TTACCGACAGCCCATCCT | TGAAAAATGCTGGTTGAATAGTAAAA | Fig-7 |
| Aatf | CTGAGGGACTTGGATGAGGA | GCTGGTTTTCCGCTCTATGA | Fig-7 |
|  |  |  |  |
| **Adenoviral vectors** | ***Ref Sequence*** | ***Source / reference*** | ***Data (Figs)*** |
| h-GCKR | BC130481 (446P) | ADV-209743 (Vector Biolabs,PA) | S1,F1,S3,S4,F2 |
| h-GCKR-446L | BC130481 (446L) | Custom (Vector Biolabs,PA) | S1,F1,S3,S4,F2 |
| m-Gckr-t2 | BC012412 (446P) | ADV-259984 (Vector Biolabs,PA) | S1, |
| m-Gckr-t2-446L | BC012412 (P446>L) | ADV-259984 (Vector Biolabs,PA) | S1 |
| m-Gckr-t1 | XM_006503881.3 | Custom (Vector Biolabs,PA) | S1,F1,S3,S4,F2 |
| m-tGckr-1-446L | XM_006503881.3 (446L) | Custom (Vector Biolabs,PA) | S1,F1,S3,S4,F2 |
| m-Gckr-X1 | XM_006503882 | Custom (Vector Biolabs,PA) | S1 |
| m-Gckr-X2 | XM_006503883 | Custom (Vector Biolabs,PA) | S1 |
| h-SIRT2 | BC003547 | ADV-1519 (Vector Biolabs,PA) | S3 |
| r-Gck | GenBank M25806.1 | Becker et al. (1996) | F1,S4,F2 |
|  |  |  |  |
| **Mouse Lines** | ***Name*** | ***International Identifier /Reference*** | **Figs** |
| Gckr DEL | GCKR-DEL1262-EM1-B6N | C57BL/6NTac-Gckr^em1(IMPC)H^/H | F1,S1 |
| Gckr P446>L | GCKR-P446L-EM1-B6N | Codner et al. (2018) | F1,S1,F2-F7,S5 |
|  |  |  |  |
| **Antibodies** | ***Vendor*** | ***Catalogue reference / Identifier*** | Figs |
| GKRP WB | AstraZeneca | Az680 or 681 | F1,S1,F3,S3 |
| GK WB | Protein Tech | 15629-1-AP | F1,F3 |
| GKRP WB | Santa Cruz | sc6340 | F5 |
| GK WB | AstraZeneca | Az600 | F5 |
| Gapdh WB | Protein Tech; Hytest | 60004-1-lg; ABIN153387 | F1,S1 |
| Beta-Actin WB | Protein Tech; Abcam | 69009-1-lg; ab8226 | F3 |
| GKRP IHC | Santa Cruz | sc6340 | F1,S1,F3,S4 |
| GK IHC | Santa Cruz | sc7908 | F1,S1, |
| GK IHC | Protein Tech | 15629-1-AP | F1,F3,S4 |
| HMGCR IHC | Protein Tech | 13533-1-AP | F6 |

| **Database** | **Gckr** | **DNA** | **nt** | **Protein** | **aa** |
| --- | --- | --- | --- | --- | --- |
| NCBI | Human | NM_001486.4 | 2189 | NP_001477.2 | 625 |
| NCBI | Rat | NM_013120.2 | 2296 | NP_037252.1 | 627 |
| NCBI | Transcript/Isoform 1 | NM_001374741.1 | 2140 | NP_001361670.1 | 623 |
| NCBI | Transcript/Isoform 2 | NM_144909.2 | 2032 | NP_659158.1 | 587 |
| NCBI | X1 | XM_006503882 | 2383 | XP_006503945.1 | 605 |
| NCBI | X2 | XM_006503883 | 2281 | XP_006503946.1 | 571 |
| NCBI | X1 (previous) | XM_006503881.4 | 2437 | XP_006503944.1 | 623 |
| NCBI | Canonical (previous) | NM_144909.1 | 2024 | NP_659158.1 | 587 |
| ENSEMBL | 201 | ENSMUST00000072228.8 | 2014 | ENSMUST00000072228.8 | 587 |
| ENSEMBL | 202 | ENSMUST00000201166.3 | 2272 | ENSMUST00000201166.3 | 623 |

Becker TC, Noel RJ, Johnson JH, Lynch RM, Hirose H, Tokuyama Y, Bell GI, Newgard CB. Differential effects of overexpressed glucokinase and hexokinase I in isolated islets. Evidence for functional segregation of the high and low Km enzymes. J Biol Chem. 1996;271:390-4.

Codner GF, Mianné J, Caulder A, Loeffler J, Fell R, King R, Allan AJ, Mackenzie M, Pike FJ, McCabe CV, Christou S, et al. Application of long single-stranded DNA donors in genome editing: generation and validation of mouse mutants. BMC Biol. 2018;16:70.
